# Mono-ubiquitylated ORF45 Mediates Association of KSHV Particles with Internal Lipid Rafts for Viral Assembly and Egress

**DOI:** 10.1371/journal.ppat.1005332

**Published:** 2015-12-09

**Authors:** Xin Wang, Nannan Zhu, Wenwei Li, Fanxiu Zhu, Yan Wang, Yan Yuan

**Affiliations:** 1 Institute of Human Virology and Ministry of Education Key Laboratory of Tropical Disease Control, Zhongshan School of Medicine, Sun Yat-Sen University, Guangdong, China; 2 Department of Microbiology, University of Pennsylvania School of Dental Medicine, Philadelphia, Pennsylvania, United States of America; 3 Department of Biological Sciences, Florida State University, Tallahassee, Florida, United States of America; 4 Guanghua School of Stomatology, Guangdong Provincial Key Laboratory of Stomatology, Sun Yat-Sen University, Guangzhou, Guangdong, China; University of Southern California, UNITED STATES

## Abstract

Herpesviruses acquire their envelope by budding into the lumen of cytoplasmic membrane vesicles. This process is initiated by component(s) on viral particles, which recognize the budding site where the viral glycoproteins are present and recruit cellular cargo transport and sorting machinery to the site to complete the budding process. Proteins in the tegument layer, connecting capsid and envelope, are candidates for the recognition of budding sites on vesicle membrane and induction of budding and final envelopment. We examined several outer and matrix tegument proteins of Kaposi’s sarcoma-associated herpesvirus (KSHV) and found that ORF45 associates with lipid rafts (LRs) of cellular membrane. LRs are membrane micro-domains, which have been implicated as relay stations in intracellular signaling and transport including viral entry and virion assembly. The ability of ORF45 to target LR is dependent on the mono-ubiquitylation of ORF45 at Lys297 as the mutation at Lys297 (K297R) abolished LR-association of ORF45. The K297R mutation also impairs ORF45 and viral particle co-localization with trans-Golgi network and endosomes, but facilitates ORF45 and viral particles co-localizing with lysosomes. More importantly, the recombinant KSHV carrying ORF45 K297R mutant (BAC-K297R) was found severely defective in producing mature and infectious virion particles in comparison to wild type KSHV (BAC16). Taken together, our results reveal a new function of KSHV tegument protein ORF45 in targeting LR of host cell membrane, promoting viral particles co-localization with trans-Golgi and endosome vesicles and facilitating the maturation and release of virion particles, suggesting that ORF45 plays a role in bringing KSHV particles to the budding site on cytoplasmic vesicle membrane and triggering the viral budding process for final envelopment and virion maturation.

## Author Summary

Most enveloped viruses acquire their envelope membrane by budding into cellular membrane. Although viruses utilize cellular cargo transport and sorting machinery for their budding into luminal vesicles or at plasma membrane, the budding process is initiated and controlled by viral component(s). For some viruses, a single viral protein possesses all the information necessary for virus budding to generate virus-like particles (VLP) in the absence of other viral components. Herpesviruses also gain their membrane envelope through budding into cytoplasmic membrane vesicles (trans-Golgi and endosome vesicles) and the underlying mechanism is much less understood in comparison to that of RNA viruses. We searched for herpesviral components that initiate or orchestrate viral budding in final envelopment and egress. We found that a tegument protein of Kaposi’s sarcoma-associated herpesvirus (KSHV), namely ORF45, associates with lipid rafts of cell membranes and this association is regulated by a mono-ubiquitylation of ORF45 at Lys297. The ubiquitylated ORF45 determines the association with trans-Golgi and endosome vesicles and facilitates the final virion maturation and production. These data suggest that ORF45 may function in recognition of budding site and recruiting cellular transport and sorting machinery for KSHV budding, envelopment and mature virion release.

## Introduction

Most enveloped viruses acquire their envelope membrane by budding into cellular membranes, either plasma membrane or intracellular membrane. When viral budding occurs at the plasma membrane, virions (such as influenza virus) are released into extracellular space. But for many other viruses (including herpesviruses), budding occurs on intracellular membranes, resulting in temporary accumulation of viral particles in the lumen of cellular organelles (such as endoplasmic reticulum [ER], Golgi network and endosomes). The viral particles are released through a subsequent transport of virus-filled vesicles towards the cell surface followed by their fusion with the plasma membrane (Reviewed in [1]).

Viral budding is topologically similar to the process of cellular cargo transport and sorting into luminal vesicles. Thus it is not surprising that viruses are often found to usurp the cellular machinery for viral budding and egress. However, the driving forces for viral budding are still viral components that initiate and orchestrate the process. For example, HIV-1 relies on host ESCRTs for release from cells, but HIV-1 Gag protein controls the process by directly binding TSG101 or Alix and recruiting ESCRT components to sites of virus budding (Reviewed in [2]). In addition, ESCRTs are known to sort ubiquitylated cargo proteins to endosomes and it is believed that conjugating ubiquitin to cargo proteins serves as a signal for ESCRT-dependent entry and sorting in endosomes [3, 4]. Ubiquitylation also plays a role in HIV-1 budding and the contribution of ubiquitylation of HIV-1 Gag to TSG101 recruitment and ESCRT-dependent virus budding has been documented [5–7].

In contrast to retroviruses in which the budding process and underlying mechanism have been well understood, much less is known about herpesvirus budding and egress. Currently, the sketchy model for *Alphaherpesvirinae* assembly and egress is the envelopment-deenvelopment-reenvelopment model [8–12]. In this model, mature HSV-1 nucleocapsids assemble in the nucleus, then undergo a process of primary envelopment through the inner nuclear membrane into the perinuclear space. This is followed by deenvelopment at the outer nuclear membrane, recruitment in the cytoplasm of tegument onto the capsid before secondary envelopment and acquisition of envelope membrane containing viral glycoproteins (cellular proteins as well) by budding into trans-Golgi network vesicles. Fully assembled virions are finally released by exocytosis. However, the details of herpesviral particle assembly and egress are largely not understood. In the final envelopment, how does a herpesvirus determine and recognize the budding site where viral glycoproteins and other necessary cellular proteins are present? How do viral proteins participate in or orchestrate the process for budding and egress? What cellular machinery does a herpesvirus harness and utilize? Is ubiquitylation of viral proteins required for the viral budding? These questions remain elusive.

Herpesvirus capsids, after nuclear egress, acquire tegument layer in the cytoplasm before being engaged in final envelopment and budding process. The tegument is subdivided into the inner and the outer tegument based on association with capsids as well as physical position in the capsid-tegument particles [8, 13, 14]. The outer tegument proteins are believed to provide a surface for interacting with cellular membrane trafficking and sorting machinery and are candidates for initiating or regulating the herpesvirus budding and envelopment process. We have identified a dozen viral proteins in the tegument layer of Kaposi’s sarcoma-associated herpesvirus (KSHV) [15] and characterized the protein-protein interaction network among the tegument proteins as well as between the tegument and capsid and between the tegument and glycoproteins [16]. This tegument protein interaction network serves as a roadmap that allows us to predict functions of some of the tegument proteins in KSHV particle assembly and egress. In this study, we chose several KSHV tegument proteins that are located in the outer tegument layer or serve as matrix protein in the tegument to investigate their potential involvement in KSHV budding and egress. ORF45, a KSHV outer tegument protein, was found to interact with lipid rafts of cell membrane and contribute to KSHV budding and egress. Interestingly the association of ORF45 with lipid rafts and KSHV budding proceeding is dependent on monoubiquitylation of ORF45 at Lys297, suggesting that ORF45 may serve as an organizer for virion particle lipid raft association, budding into luminal vesicles and final envelopment of KSHV.

## Results

### Lipid rafts are crucial for KSHV egress

**Fig 1:**
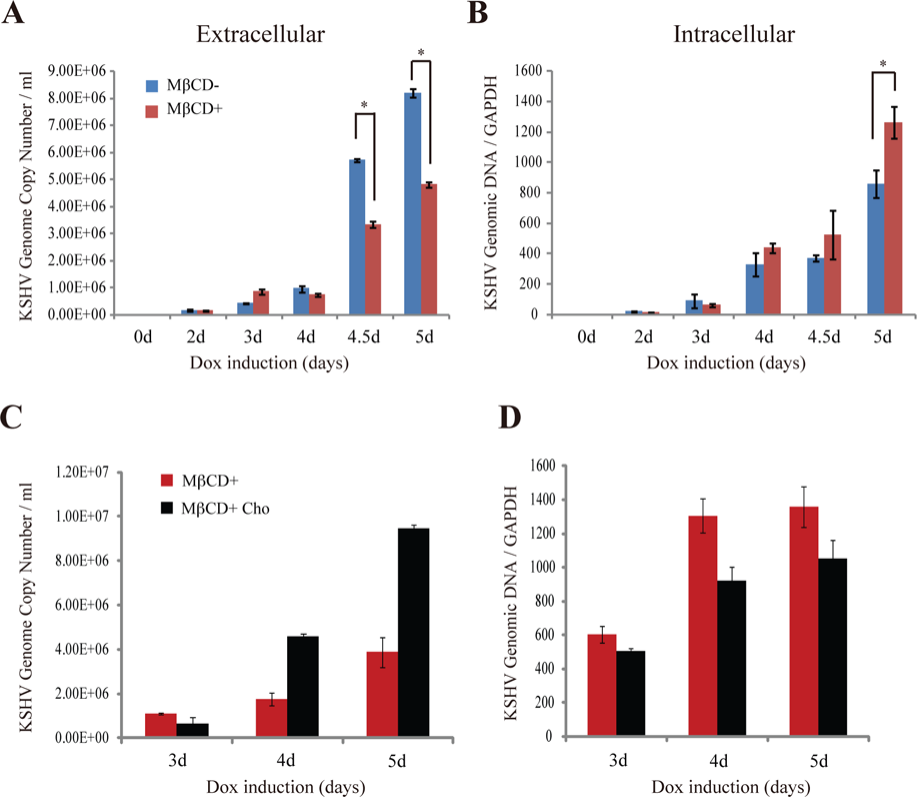
Lipid rafts are required for KSHV egress. iSLK.219 cells that carry latent KSHV genomes were cultured in 24-well plate and lytic replication of KSHV was induced by Doxycycline (Dox) for indicated days in the absence and presence of MβCD (1 mM). Extracellular virion DNA copy number (A) and intracellular viral genomic DNA (B) were quantitated by qPCR as described in Materials and Methods. (*, *p*< 0.05). After Dox induction and MβCD treatment for 1 day, water soluble cholesterol (Cho) was complemented to the iSLK.219 cell for indicated days. Extracellular virion DNA (C) and intracellular viral genomic DNA (D) were determined as above.

Enveloped viruses acquire their envelope by budding through a cellular membrane. Since the viral budding process appears to mechanistically resemble the formation of cellular vesicles, the virus may usurp cellular membrane components but under the control of viral component(s). To investigate the detailed process and underlying mechanism of herpesviral budding and final envelopment, we first asked if the viral budding and envelopment take place in lipid rafts (LRs) which are known to be important for cellular membrane vesicle formation. Toward this end, we examined the importance of LRs in KSHV virion production. KSHV lytic replication was induced in iSLK.219 cells by treating the cells with doxycycline (Dox) and sodium butyrate (NaB). The induced cells were treated with 1 mM methyl-beta-cyclodetrin (MβCD), which removes LR scaffolding component cholesterol from membrane and disrupts the integrity of LR. At this concentration (1 mM), MβCD did not show any adverse effect on cell viability and microtubule organization (S1 Fig). The effect of MβCD on KSHV virion production was determined by comparing the amounts of virions released to media as well as viral DNA replication in the cells in the presence and absence of MβCD. Extracellular virion DNA was extracted from viral particles in cell culture at different times post-induction to determine the released virions in the media. Meanwhile intracellular viral genomic DNA was also extracted from induced iSLK.219 cells to measure intracellular KSHV genomic DNA content. The extracellular and intracellular viral DNA copy number was examined by qPCR. The results showed that MβCD treatment resulted in significant reduction in production of KSHV virions in comparison to the control without MβCD treatment (Fig 1A). However, MβCD treatment did not affect viral DNA replication until five day post-induction. On the fifth day, the intracellular KSHV genomic DNA in MβCD-treated cells appears to be more than that in untreated cells, probably due to failure to release progeny viral particles into medium from MβCD-treated cells allowing viral genomic DNA accumulation inside the cells (Fig 1B). Similarly, addition of lovastatin (2 μM), which is an inhibitor of cholesterol biosynthesis, to the culture media also reduced KSHV virion production (S2 Fig).

To further confirm the role of cholesterol-rich LRs in KSHV virion production, we tested if addition of cholesterol can revert the MβCD effect by complementing water-soluble cholesterol (400 μg/mL) to the MβCD-treated iSLK.219 cells. Result showed that addition of water-soluble cholesterol efficiently rescued MβCD-caused reduction of KSHV virion production and enhance the virus release (Fig 1C and 1D). These data indicate that LRs play a crucial role in KSHV assembly and egress.

### ORF45 interacts with lipid rafts

**Fig 2:**
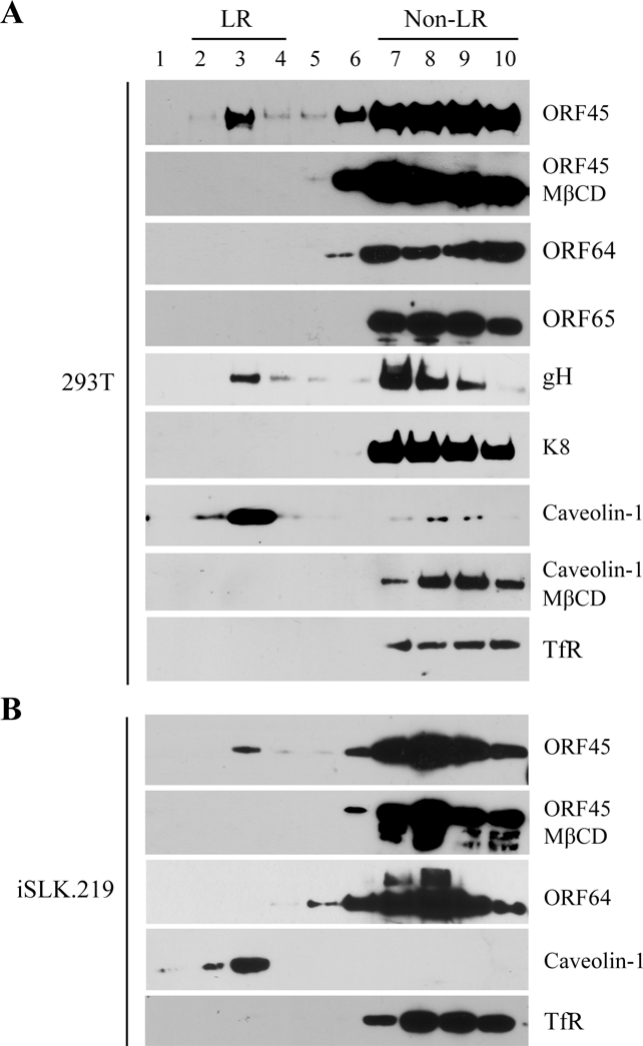
Association of ORF45 with lipid rafts. (A) HEK293T cell was transfected with expression vector of KSHV proteins ORF45, ORF64, ORF65, gH and K8, respectively. Forty-eight hour post-transfection, the cells were subjected for membrane flotation assay as described in Materials and Methods. Ten fractions (line 1–10) were collected from the top to the bottom of the ultracentrifuged sucrose gradients and analyzed by Western blotting with specific antibodies as indicated. Caveolin-1 and TfR served as controls that defined the detergent resistant membrane (DRM) or LR (lane 2–4) and non-LR (lane 7–10) fractions. (B) The association of ORF45 with LRs in the context of virus was examined using iSLK.219 cells induced by Dox for 48 hrs. The membrane flotation assay was performed to analyze the LR-localization of ORF45 and ORF64 with mouse anti-ORF45 and anti-ORF64 antibodies. The ORF45 transfected 293T and induced iSLK.219 cells were treated with MβCD (1 mM) for 24 hours and the cells were subjected for membrane flotation assay as described above.

After nuclear egress, herpesviral capsids acquire tegument proteins in the cytoplasm and the tegumented capsids target internal membranes (such as Golgi apparatus and endosomes) for budding [8]. We rationalize that the membrane targeting of viral particles might be mediated by an outermost tegument protein or proteins on the viral particles. To identify the component(s) that contributes to the process, several KSHV tegument proteins were examined for capabilities to interact with LRs using a membrane flotation assay. This assay allows for isolation of the detergent resistant membrane (DRM), which generally consists of LR domains as the tight packing of LRs enables them to resist nonionic detergent solubility at low temperature, and detection of DRM-association proteins. 293T cells were transfected with expression vectors for the tegument proteins. Forty-eight hour post-transfection, the membrane fraction was prepared and treated with 0.5% Triton X-100 on ice and then layered at the bottom of a sucrose gradient. After ultracentrifugation, LRs floated up to the upper layer (less dense sucrose layer) of the gradient. Fractions were collected from the top to the bottom and analyzed by Western blotting for tegument proteins. The result showed that ORF45, an abundant outer tegument protein [15], was detected in the fractions of DRM where the LR marker caveolin-1 was located (Fig 2A). When cells were treated with MβCD that disrupts LRs, ORF45, as well as caveolin-1, were no longer detected in the LR fractions (Fig 2A). In contrast, ORF64, a major tegument matrix protein, was not detected in DRM fractions when expressing alone in 293T cells. As expected, glycoprotein H (gH) was present in DRM fractions, but nuclear protein K8 was not (Fig 2A).

In addition, the ORF45 association with LRs was also tested in the context of KSHV. iSLK.219 cells, that carry latently infected KSHV and doxycycline inducible RTA, were induced for lytic viral replication. Forty-eight hour post-induction the cells were lysed and the DRM membrane fractions were analyzed with membrane flotation assay. As shown in Fig 2B, ORF45 was found to be associated with LRs and detected in the DRM factions.

**Fig 3:**
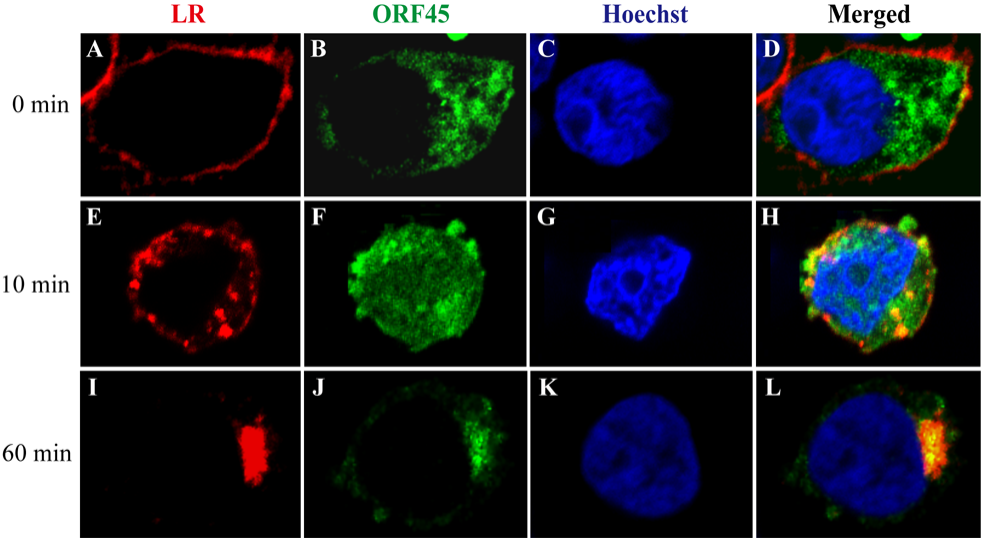
Colocalization of ORF45 with dynamic lipid rafts. HeLa cells were transfected with the expression vector of GFP-tagged ORF45. Forty-eight hour post-transfection, LRs in live cells were labeled with CTB-555 at 4°C for 15 min. After washing with pre-cooled DMEM (0 min), cells were incubated at 37°C for 10 min and 60 min, respectively, and then fixed intermediately. The nuclei were stained by Hoechst 33258 (blue). Subcellular localization of GFP-tagged ORF45 (green) and LRs (red) were examined under a Zeiss LSM780 confocal laser scanning system (63×oil). The merged image is also shown.

To determine if ORF45 colocalizes with LRs in cells, we labeled LRs with Fluor Alexa-555 conjugated cholera toxin subunit B (CTB-555), which can specifically bind LR marker ganglioside GM1 protein in live cells [17], in HeLa cells transiently transfected with GFP-tagged ORF45. At the beginning of staining, CTB-555 bound GM1 on the plasma membrane (Fig 3, upper panels; S1 Video). Time-lapse fluorescent microscopy was imaged and video-recorded. After 10 min, CTB-GM1 complexes were endocytosed into the cytoplasm (internal membranes such as endosome or Golgi apparatus) (Fig 3, middle panels; S1 Video) and after 60 min labeled GM1 was enriched in a compartment in the cytoplasm in the outer periphery of the nucleus (Fig 3I; S1 Video). The subcellular localization of ORF45 was monitored with GFP. The result showed the partial colocalization of LR marker GM1 and ORF45 at 10 min and complete overlapped after 60 min, suggesting that ORF45 colocalizes with LRs in cells (Fig 3L). In order to confirm the colocalization of ORF45 with LRs, the ORF45-transfected cells were treated with MβCD to dissemble LRs and stained with CTB-555. The results showed that the distribution of GM1 positive LR was different from that of control cells (no MβCD-treatment) and the internalization of CTB-GM1 complex was suppressed (S2 Video).

### Lys297 is crucial for LR-localization of ORF45

**Fig 4:**
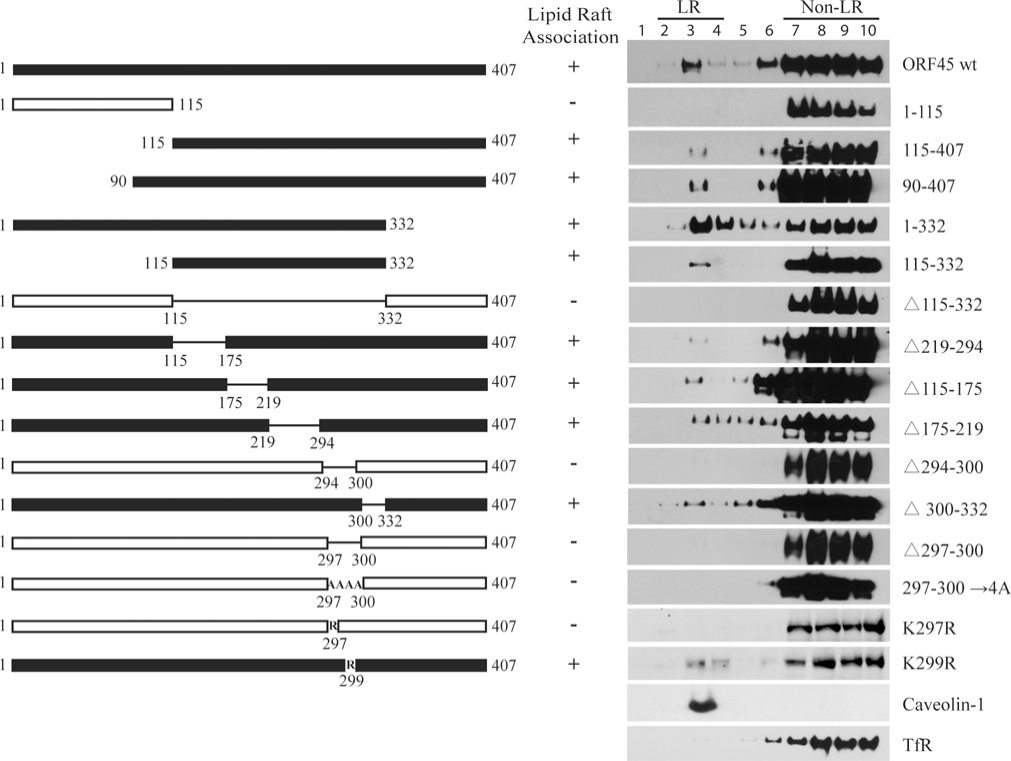
Mapping of ORF45 for LR-localization motif. A series of ORF45 truncation, deletion or amino acid substitution mutants were constructed as indicated. The expression vectors of wild type ORF45 and mutants (pCMV-3tag-1/mutant) were introduced into 293T cells, respectively. Forty-eight hour post-transfection, the cells were subjected to membrane flotation assay followed by Western blotting with anti-Flag antibody to detect the LR-association of each ORF45 mutation. Caveolin-1 and TfR served as controls that define the LR (lanes 2–4) and non-LR (lane 7–10) fractions. The ORF45 mutants that retain LR targeting ability were marked with black bar and “+” signs, while the mutants that lost LR targeting ability were marked with hollow bars and “-” signs.

To understand how ORF45 targets LRs, we mapped the protein for domain(s) that are important for LR association. A series of truncation, deletion and point mutants of ORF45 were constructed and assayed for LR targeting in 293T cells. The results narrowed down the LR-association domain to the amino acid residues of 297–300 in ORF45. Deletion of these four amino acids or mutation of them to four alanines (AAAA) abolished the ability of ORF45 to target LRs (Fig 4). To further assess the contribution of each of the amino acids to the LR targeting, the lysine at 297 and 299 residues were mutated to arginine, respectively. As results, the mutation at lysine 297 (K297R) led to complete abolishment of LR binding of ORF45, while the mutation at lysine 299 (K299R) showed little effect on LR association (Fig 4), indicating that the lysine at 297 is crucial for ORF45 LR association. Although the motif of 297–300 residues has been identified as a nuclear localization signal (NLS) of ORF45 [18], neither the mutation K297R nor K299R affects the nuclear localization of ORF45 (S3 Fig).

### Ubiquitylation of Lys297 modulates LR localization of ORF45

**Fig 5:**
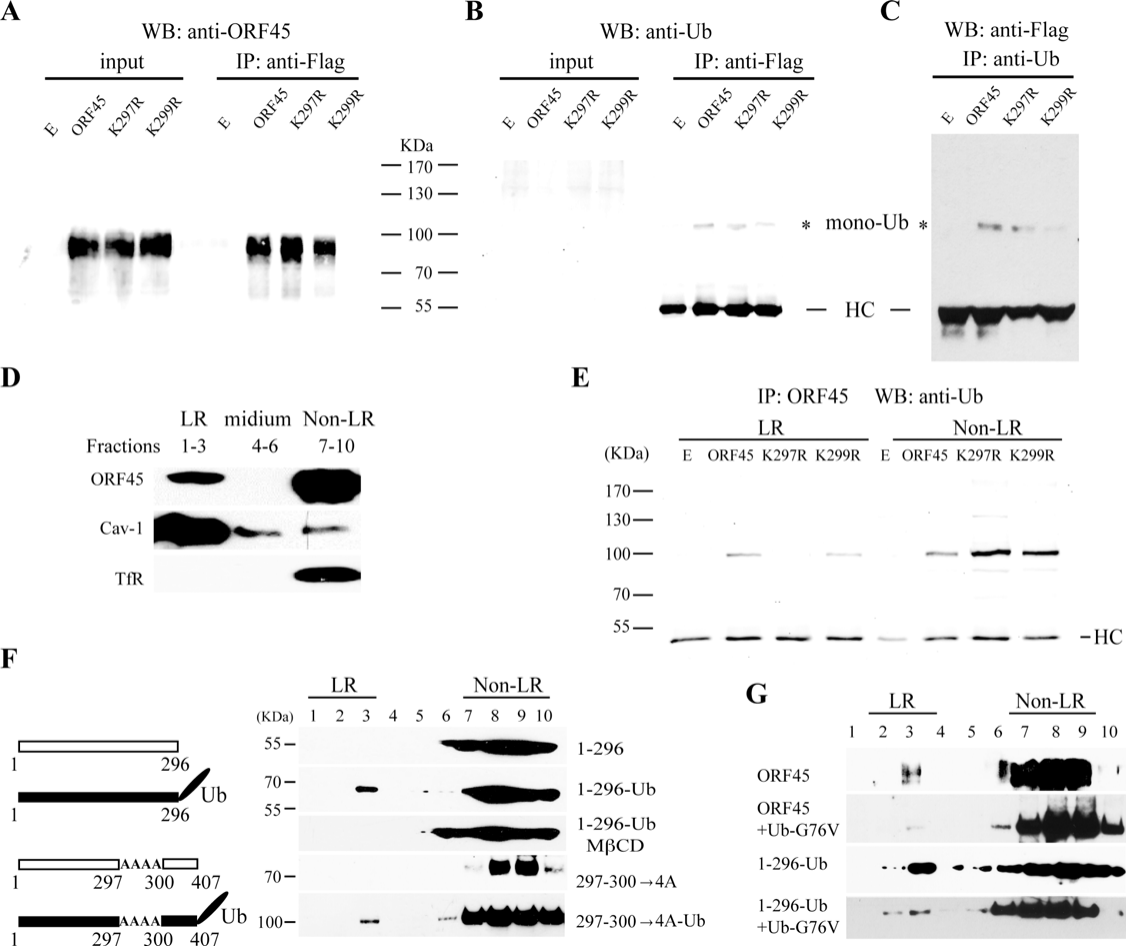
Ubiquitylation at lys297 mediates ORF45 LR-localization. Flag-tagged ORF45 and its Lys297 and Lys299 mutants (K297R and K299R) were expressed in 293T cells and immunoprecipitated with anti-Flag antibody. The input and immunoprecipitated samples were resolved on SDS-PAGE followed by Western blotting with anti-ORF45 (A) and anti-ubiquitin (anti-Ub) antibodies (B), respectively. The reverse immunoprecipitation was also performed with anti-Ub antibody followed by Western blotting with anti-Flag antibody (C). The LR (detergent resistant membrane or DRM) and non-LR fractions were separated using membrane flotation assay on a sucrose gradient as shown in Fig 2. After ultracentrifugation, the LR fractions (the top 3 fractions), non-LR (the bottom 4 fractions), and the fractions in between (the middle 3 fractions) were collected and analyzed with anti-ORF45 antibody as well as antibodies for LR and non-LR markers for confirmation (D). The LR and non-LR fractions were immunoprecipitated with anti-ORF45 antibody followed by Western blotting with anti-Ub antibody (E). To demonstrate the importance of mono-ubiquitylation of ORF45, two ORF45 mutants (1–296 amino acid and 297–300→4A) and their ubiquitin-conjugated fragment (1-296-Ub and 297–300→4A-Ub) were constructed (the left in panel F) and introduced into 293T cells. The cells were subjected to membrane flotation assay (the right in panel F). Black bars on the left indicate the ORF45 fragment capable of targeting LRs, while the hollow bars indicates the fragment that did not localize with LRs (the left in panel F). Unconjugatable ubiquitin G76V mutant expression vector were introduced into cells with ORF45 and 1-296-Ub vectors. The effect of ubiquitin G76V on LR targeting was analyzed by membrane flotation assay (G).

It has been found that the lysine in NLS often serves as an ubiquitin acceptor site, such as in p53 [19] and Matrix protein of Nipah virus [20]. We wondered if the lysine 297 in ORF45 is a potential ubiquitin acceptor and if the ubiquitylation of ORF45 at this lysine is required for LR targeting. To address this question, we first examined the ubiquitylation of ORF45 and mutants in cells. Flag-tagged wild type ORF45 and two lysine mutants (K297R and K299R) were expressed in 293T cells and immunoprecipitated from the whole cell lysates with an anti-Flag antibody. The precipitates were analyzed by Western blotting with anti-ORF45 (Fig 5A) and anti-ubiquitin (anti-Ub) antibodies (Fig 5B), respectively. The ubiquitylated ORF45 was detected with the molecular weight of approximate 10 kDa above that of ORF45, suggesting that ORF45 could be mono-ubiquitylated. The K297R and K299R mutants could also be mono-ubiquitylated (Fig 5B). We also performed a reciprocal immunoprecipitation experiment to confirm the mono-ubiquitylation of ORF45 by immunoprecipitating the cell lysates with anti-Ub antibody followed by Western blotting with anti-Flag antibody. Mono-ubiquitylated ORF45 and derivatives were detected (Fig 5C). Then, we used the same procedure to examine the mono-ubiquitylated ORF45 in LRs. LR and non-LR fractions of ORF45-transfected 293T cells were separated using membrane flotation procedure and confirmed by a Western analysis of the fractions for LR markers (Caveolin-1 for LRs and TfR for non-LRs) as well as ORF45 (Fig 5D). Both LR and non-LR fractions were subjected to immunoprecipitation with anti-ORF45 antibody, respectively and Western blotting with anti-ubiquitin antibody. Results showed that although mono-ubiquitylated forms of ORF45, K297R and K299R were detected in the non-LR fraction, mono-ubiquitylated K297R mutant was not detected in the LR fraction (Fig 5E). It is possible that ORF45 can be mono-ubiquitylated at different lysine residues (including Lys297, Lys299 or others), but only mono-ubiquitylation at Lys297 mediates ORF45 targeting of LRs.

To confirm the importance of mono-ubiquitylation at Lys297 in ORF45 LR localization, two lines of experiment were performed. First, we generated a chimeric protein of an ubiquitin-conjugated ORF45 fragment. The ORF45 truncation 1–296 aa was shown to fail to associate with LRs, however, conjugation of an ubiquitin moiety at the carboxyl terminus of 1–296 aa fragment conferred the ability of the truncated ORF45 fragment to interact with LRs (Fig 5F). Furthermore, addition of an ubiquitin moiety to the carboxyl terminus of ORF45(297-300-4A) mutant also render this mutant ability to associate with LRs (Fig 5F). Second, we examined if inhibition of ORF45 ubiquitylation by introducing unconjugatable ubiquitin (G76V) into cells suppresses ORF45 association with LRs. Ubiquitin-G76V expression vector was co-transfected with wild type ORF45 or the fragment 1-296-Ub respectively and the effect of unconjugatable ubiquitin on LR association was analyzed by membrane flotation as described above. As shown in Fig 5G, the LR association of ORF45 was greatly reduced in the presence of G76V. In contrast, G76V did not affect the association of the 1-296-Ub fragment with LRs, suggesting that covalent conjugation of ubiquitin to ORF45 is necessary for LR targeting. Furthermore, unconjugated ubiquitin G76V inhibited colocalization of ORF45 with LRs but had no effect on 1-296-Ub colocalization with LRs (S4 Fig). Taken together, we conclude that the mono-ubiquitylated modification at Lys297 is crucial for LR-localization of ORF45.

### Lys297 contributes to ORF45 colocation with LR and Golgi markers

**Fig 6:**
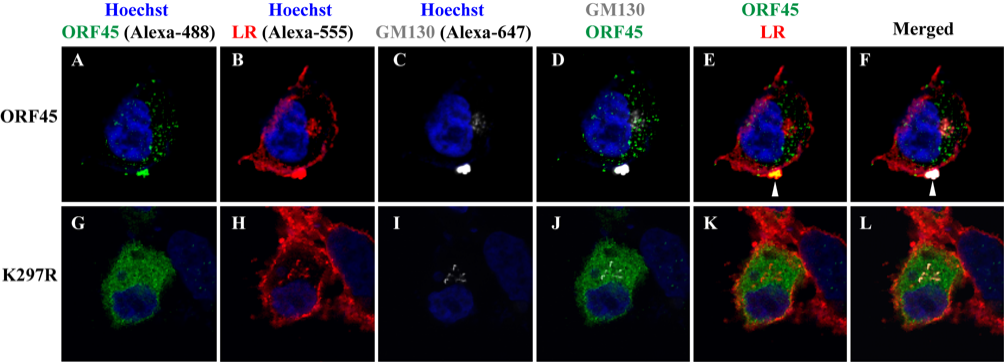
ORF45 colocalizes with LR and Golgi markers but K297R mutant does not. iSLK cells were transfected with ORF45 (A-F) or K297R mutant (G-L), respectively. Forty-eight hour post-transfection, cells were subjected to triple-labeled IFA using CTB-555, rabbit polyclonal anti-GM130 and mouse monoclonal anti-ORF45 as described in Materials and Methods. Alexa-488 conjugated anti-mouse IgG (green) and Alexa-647 conjugated anti-rabbit IgG (white) were used as the respective secondary antibodies. The nuclei were stained by Hoechst (blue). The two or three channels merged images are shown. The white arrows mark the sites of colocalization.

The effect of K297R mutation on LR targeting of ORF45 was further analyzed using immunofluorescence assay (IFA). ORF45 and its K297R mutant were ectopically expressed in iSLK cells. The subcellular localization of ORF45 or K297R relative to LRs and Golgi membrane network were examined by triple-labeled IFA. ORF45 and K297R were labeled with a mouse anti-ORF45 antibody and stained with Alexa-488 (green) conjugated goat anti-mouse IgG. LRs were stained with CTB-555. The Golgi apparatus was visualized with the Golgi marker GM130, a Golgi matrix protein, labeled by Alexa-647 (defined as white) conjugated goat anti-rabbit IgG. Wild type ORF45 was found to colocalize with Golgi (GM130) and LR markers (Fig 6D and 6E, pointed by white arrowheads). However in the cells transfected with the K297R mutant, the mutated ORF45 was diffused throughout the cytoplasm and did not appear to colocalize with either LR or Golgi markers (Fig 6J and 6K), though LR colocalized with the Golgi marker in all transfected cells.

**Fig 7:**
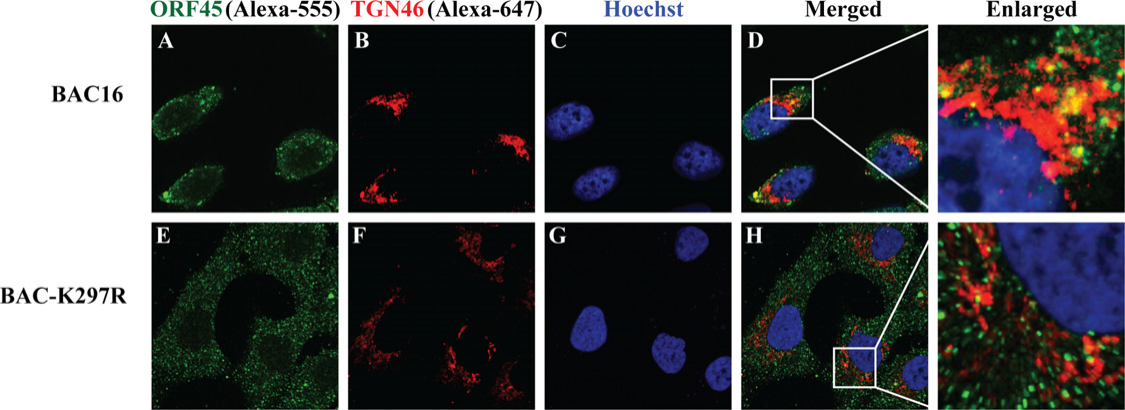
ORF45 colocalizes with trans-Golgi network but K297R mutants does not in the context of virus. Cells carrying BAC-cloned KSHV (iSLK-BAC16) and ORF45-K297R mutant recombinant virus (iSLK-BAC-K297R) were induced with Dox (3 μg/ml) for lytic KSHV replication. Three days post-induction, cells were stained with mouse monoclonal anti-ORF45 (green) and rabbit polyclonal anti-TGN46 (red). The nuclei were stained by Hoechst (blue). The white boxes within the merged panels are shown as enlarged pictures for the details of colocalization.

Then we characterized the LR targeting property of ORF45 as a tegument protein in a viral particle in Golgi apparatus and the effect of K297R mutation on it. Toward this end, we examined colocalization of ORF45 with *trans*-Golgi network in the context of virus. We constructed a K297R mutant recombinant KSHV using a BAC-cloned KSHV (BAC16) [21] and the Red-mediated recombination procedure [22] (S5 Fig). The K297R mutant virus (BAC-K297R) was reconstituted by transfecting iSLK cells followed by hygromycin selection. Lytic replication of BAC-K297R and wild type BAC16 in iSLK cells were induced by treatment with Dox for 72 hours. The cell lines carrying BAC16 and BAC-K297R express GFP, which occupied Alexa-488 channel when observe under confocal microscope but allowed us to analyze ORF45 and TGN46 with Alexa-555 conjugated anti-mouse IgG (green for ORF45) and Alexa-647 conjugated anti-rabbit IgG (Red for TGN46) (Fig 7). Consistent with the result of ORF45-transfected cells, OR45 in the cells with wild type KSHV was found to colocalize with the TGN marker (TGN46) in the cytoplasm (Fig 7A–7D). In contrast, the K297R mutant did not colocalize with TGN46 (Fig 7E–7H).

### KSHV virions mature through the Golgi network and Golgi-derived vesicles, but BAC-K297R mutant virus fails to do so

**Fig 8:**
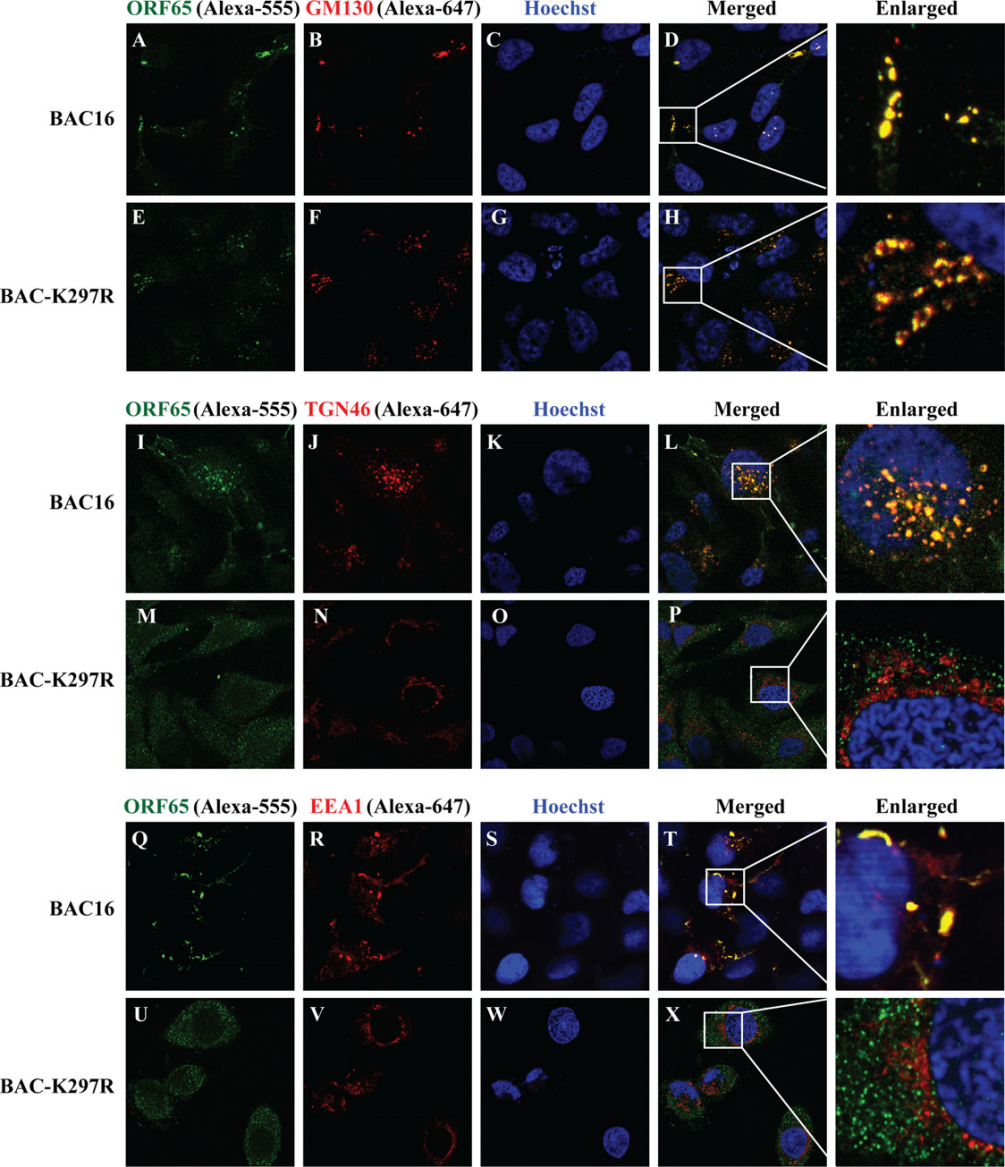
Localization of KSHV particles in iSLK-BAC16 and iSLK-BAC-K297R cells. iSLK-BAC16 and iSLK-BAC-K297R cells were induced with Dox (3 μg/ml) for lytic KSHV replication. Three days post-induction the viral particles were labeled with mouse anti-ORF65 antibody, while the *cis*-Golgi, *trans*-Golgi network (TGN) and early endosomes were labeled with anti-GM130 (upper panels, A–H), anti-TGN46 (middle panels, I–P) and anti-EEA1 (lower panels, Q–X) rabbit polyclonal antibodies, respectively. The Alexa-555 conjugated anti-mouse IgG (green) and Alexa-647 conjugated anti-rabbit IgG (red) were used as the respective secondary antibodies. The channels Alexa-555 and Alexa-647 were defined in green and red color respectively in ZEN2009 and Imaris software. The white boxes within the merged panels are shown as enlarged images for the details of colocalization.

We also examined viral particles for their internal membrane location through visualizing them with an antibody against capsid protein ORF65 in iSLK-BAC16 and iSLK-BAC-K297R cell. Although ORF65 staining may detect not only capsids but also other forms of ORF65, the colocalization of ORF65 with ORF45 suggests that the ORF65 positivity in the cytoplasm represents tegumented capsid (S6 Fig). Three days post-induction, viral particles in cells were labeled with an anti-ORF65 antibody that was stained by Alexa-555 conjugated secondary antibody (defined as green in Fig 8). The organelle markers (GM130, TGN46 and early endosomal marker EEA1) were labeled by Alexa-647 conjugate secondary antibody (defined as red in Fig 8). Wild type viral particles colocalized with *cis*-Golgi marker GM130 (Fig 8D), *trans*-Golgi network marker TGN46 (Fig 8L), and early endosome marker EEA1 (Fig 8T). While in iSLK-BAC-K297R cells, the capsid can be transported at the *cis*-Golgi (Fig 8H), but failed to localize to the *trans*-Golgi network and the early endosome (Fig 8P and 8X). These data suggest that ORF45 targets LRs in the Golgi network to direct capsids budding into the Golgi-derived vesicles, which contains both TGN and endosomal markers. By losing ORF45 LR-localization function, the K297R mutant virus may attach to the *cis*-Golgi membrane (possibly mediated by other tegument proteins), but cannot pass through the Golgi complex membrane and gain its envelope.

### BAC-K297R mutant particles accumulate in lysosomes for degradation

**Fig 9:**
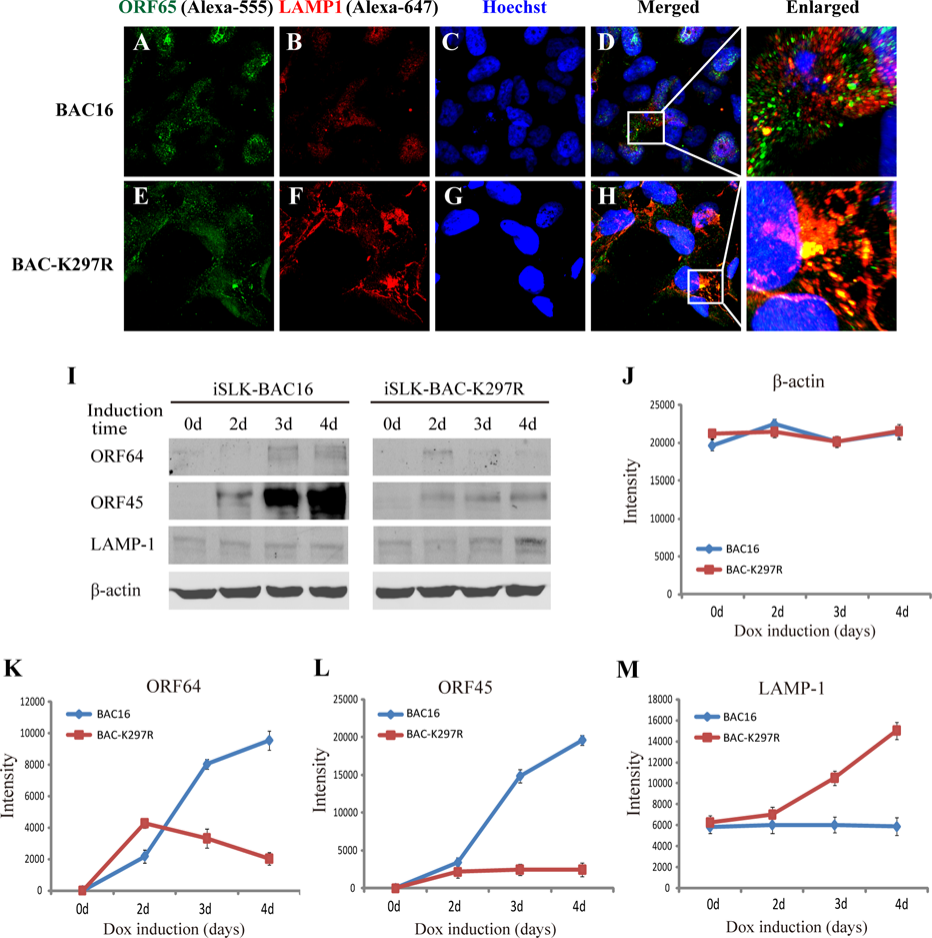
BAC-K297R mutant virus is colocalized with the lysosomes. iSLK-BAC16 and iSLK-BAC-K297R cells were induced with Dox (3 μg/ml) for 3 days for viral lytic replication. The double-labeled IFA was performed with mouse monoclonal anti-ORF65 and rabbit polyclonal anti-LAMP-1. Alexa-555 conjugated anti-mouse IgG and Alexa-647 conjugated anti-rabbit IgG were used as the respective secondary antibodies. The channels of Alexa-555 and Alexa-647 were defined in green and red, respectively. The colocalization sites were enlarged as shown (A–H). iSLK-BAC16 and iSLK-BAC-K297R cells were induced with Dox and butyrate and lysed by RIPA lysis buffer at indicated time of post-induction. The cell lysates were resolved on 8% SDS-PAGE and analyzed by Western blotting with antibodies against tegument proteins ORF64, ORF45 and lysosome marker LAMP-1, respectively. β-actin serves as a loading control (I). The intensity of each band was quantitated using ImageJ software (J–M).

Both wild type and K297R viral particles can be transported to cis-Golgi apparatus but only wild type viral particle can be detected in the trans-Golgi network. Where are the mutant viral particles ended up in the cytoplasm? We found that when iSLK-BAC16 and iSLK-BAC-K297R cells were stained with antibody against lysosome marker LAMP1, K297R mutant particles were colocalized with LAMP1, while the colocalization in wild type virus was not obvious in iSLK-BAC16 cells (Fig 9A–9H), suggesting that the mutant particles colocalized with lysosomes. Furthermore, Western analysis, shown in Fig 9I and quantitated in Fig 9M, indicates that the expression of LAMP-1 increased in iSLK-BAC-K297R cells in comparison to that in iSLK-BAC16 cells. Meanwhile, the expression of tegument proteins ORF45 and ORF64 decreased dramatically in iSLK-BAC-K297R cell on 4th day post-induction (Fig 9K and 9L). It appeared that deficiency of ORF45 LR-localization interrupts the virion egress pathway, and the mutant viral particles fail to enter the Golgi complex, but accumulate in the lysosome for degradation.

### The K297R mutation severely reduces the release of infectious progeny virions

**Fig 10:**
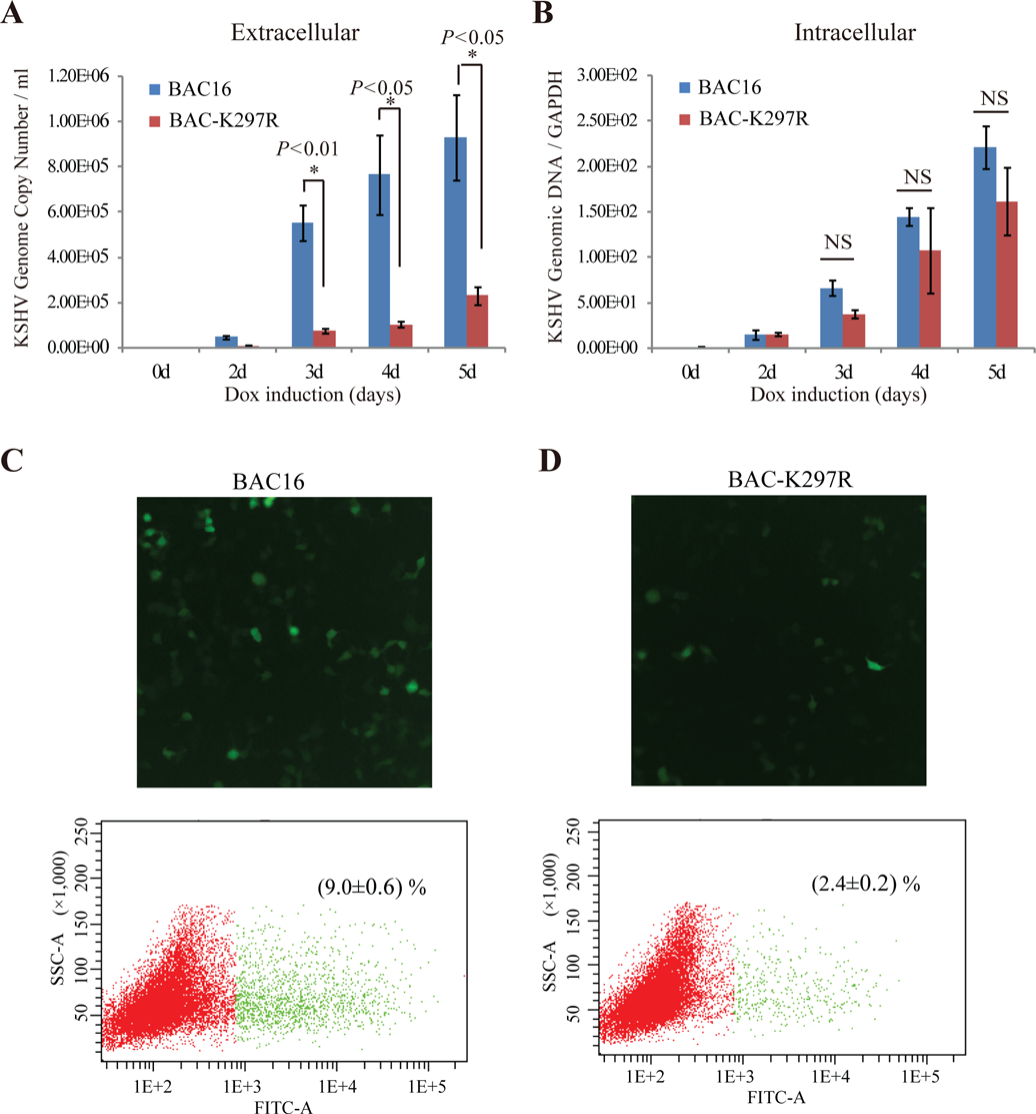
Effect of the K297R mutation on progeny virus release. iSLK-BAC16 and iSLK-BAC-K297R cells were induced with Dox and butyrate for indicated time. The extracellular virions were collected from culture media and treated with Turbo DNase I. Viral DNAs were extracted and KSHV genomic DNA copy numbers were estimated by qPCR along with external standards of known concentrations of the viral DNA with primers against the ORF73 gene (A). Intracellular KSHV genomic DNAs were extracted from harvested cells and quantitated by qPCR as above (B). (*, *p*<0.05; NS, no significant difference). (C and D) The progeny viruses were purified and concentrated 200-fold from the cell culture of iSLK-BAC16 and iSLK-BAC-K297R cells. Progeny virions with same DNA copy number of BAC16 and BAC-K297R were used to infect 293T cell as described in Materials and Methods. The infectivity was examined by GFP fluorescence using fluorescence microscopy and flow cytometry analysis. Three duplications were prepared for flow cytometry and the means ± sd of the infectivity was graphed.

At last, we examined the contribution of ORF45 targeting LR to virion assembly and release of infectious virion particles by analyzing the effect of the K297R mutation on virion production. Wild type iSLK-BAC16 (wild type) and iSLK-BAC-K297R cells were induced with Dox for five days and released virions in the media were quantitated with qPCR. iSLK-BAC-K297R released much less progeny virions than the cells carrying wild type iSLK-BAC16 viral genome (Fig 10A), while there were no significant difference in the intracellular viral genomic DNA between BAC16 and BAC-K297R (Fig 10B). Furthermore, we found that the K297R mutation not only reduced the quantity of virions released from cells, also may affect the quality of the progeny virus as the virions prepared from the medium of iSLK-BAC-K297R demonstrated a much lower infection rate in comparison to iSLK-BAC16 in the same multiplicity of infection (MOI = 100 viral genome equivalent). The infection ratio was analyzed by fluorescence microscope and flow cytometry as the infected cells expressed GFP (Fig 10C and 10D). These data suggest that BAC-K297R mutant produces fewer virion particles than wild type BAC16 and many of the particles produced by BAC-K297R are immature and non-infectious.

## Discussion

Herpesvirus assembly and egress is an important but underappreciated research area. The commonly accepted concept about herpesvirus assembly and virion production is that it is a multiple-stage process where (i) newly synthesized viral DNA is incorporated into preformed capsid in the nucleus; (ii) the nucleocapsid leaves the nucleus by first budding through the inner nuclear membrane, formatting primary enveloped virions in the perinuclear space, and the primary envelope then fuses with the outer leaflet of the nuclear membrane, thereby releasing nucleocapsids into the cytoplasm; (iii) acquisition of tegument takes place in the cytoplasm; (iv) final envelopment, including acquisition of membrane envelope with glycoproteins, occurs by budding into Golgi-derived vesicles; (iv) mature virions are released after fusion of the vesicle membrane with the plasma membrane of the cell (reviewed in [8] and [11]). However the detailed processes and the underlying mechanisms that control these processes are still elusive and some are even in contention [12]. Acquisition of membrane envelope by budding into internal membrane network is a complex but highly regulated process which involves (i) the recognition by viral particles of budding site on the internal membrane where viral glycoproteins and other necessary components are present; (ii) recruitment and assembly of cellular vesicle formation machinery; and (iii) induction of vascularization-like process that bud virions into luminal vesicles. This process and the regulation are also not fully understood.

A herpesviral virion contains one or two dozens of tegument proteins, which can be divided into inner and outer tegument proteins based on their positions and how tightly the proteins are associated with capsids [8, 13, 14]. Acquisition of tegument proteins is thought to initiate in the nucleus, then more tegument proteins are added to capsids in the cytoplasm following nuclear egress and finally at *trans*-Golgi network (TGN)-derived membranes during maturation budding [8, 9]. The complexity of the virion assembly route clearly raises the issue that different tegument proteins may be incorporated into herpesviral particles at different stages of egress. ORF45 is an outer tegument protein of KSHV, of which homologues are present only in gamma-herpesviruses [15, 23]. At the early stage of infection, ORF45 is released in the cytoplasm to antagonize host antiviral response and regulate cell signaling following envelope fusion with plasma membrane [24, 25]. During virion assembly, ORF45, as an outer tegument protein, may participate in interaction with cellular components for viral assembly and final envelopment. We reported previously that ORF45 interacts with motor molecule kinesin-2 and is responsible for transport of capid-tegument complexes along microtubules during viral particle assembly [26]. In the current study, ORF45 was found to associate with lipid rafts (LRs) and direct viral particles to the Golgi and endosome membrane for budding. A mono-ubiquitylation of ORF45 at Lys297 appears to control the LR targeting process.

LRs play a critical role in KSHV particle assembly and egress as progeny virus formation and production was significantly inhibited when membrane LRs were disrupted by cholesterol removing agents (Fig 1). This was an anticipated result for several reasons. First, herpesviral particles acquire a virion envelope by budding into cytoplasmic membranes (the Golgi apparatus and endosomes) at the site where viral glycoproteins are enriched. The viral glycoproteins are in general enriched in LRs [27] and a herpesvirus particle is expected to bud into cytoplasmic membrane at the site of LR. This notion was supported by the finding that GM1, a representative raft marker, is found incorporated into mature HHV-6 virion particles along with the viral envelope glycoproteins [28]. Second, Enveloped virus budding mechanistically resembles the formation of cellular vesicles in which lipid rafts play important roles. Thus, virus budding may also occur in LR platforms. Third, many other viruses are known to utilize LRs during budding [29–33]. Many viral glycoproteins and structural proteins target LR for viral particle assembly and egress, such as HA, NA and M2 proteins of influenza virus [29, 30], HIV Gag protein [32, 33], and HCV NS5A protein [34]. ORF45 of KSHV has been added to the growing list of the viral proteins that promote viral budding and egress by harnessing cellular LRs.

While it is believed that virus budding is driven and controlled by viral machinery, recent studies demonstrated the requirement of particular cellular functions in this process. In fact, viruses can usurp cellular machinery to promote budding and egress. The process of viral budding is topologically similar to cellular cargo recognition and multivesicular body biosynthesis process by the endosomal sorting complexes required for transport (ESCRT) machinery. The ESCRT pathway engages ubiquitylated cargos at the endosome and mediate its sorting into multivesicular bodies for degradation in lysosomes. ESCRT has been demonstrated to be used by retroviruses for viral budding and mono-ubiquitylation of Gag protein of HIV-1 is essential for ESCRT-mediated HIV-1 budding [5–7]. Our finding that ORF45 LR targeting is controlled by mono-ubiquitylation suggests that ORF45-mediated KSHV budding may also use ESCRT machinery. Further investigation on the cellular proteins or machinery required for KSHV budding and egress will answer the question whether KSHV relies on the host ESCRT machinery for viral particle budding and virion egress.

Both wild type KSHV and K297R mutant particles can colocalize with the cis-Golgi apparatus. Only wild type virus was found to colocalize with the membrane vesicles that contain both TGN and endosomal markers. This TGN46/EEA1-positive compartment (Fig 8), formed in the perinuclear area in the cytoplasm apparently induced by viral replication, may represent TGN and Golgi-derived vesicles and the site of viral budding. However, K297R mutant-containing particles failed to be detected in these TGN and Golgi-derived vesicles but were colocalized with lysosomal markers instead, suggesting that the immature viral particles may be sorted and degraded in lysosomes. We hypothesize that KSHV tegumented capsids may be brought to cis-Golgi apparatus by certain tegument protein(s) that may or may not be ORF45. Then mono-ubiquitylated ORF45 is recognized by a cellular membrane sorting complex followed by membrane invagination and budding of viral particles into Golgi-derived vesicles. In contrast, K297R mutant particles are unable to bud because of lacking Lys297 mono-ubiquitylation and as a result are engulfed by lysosomes. We may further speculate that as K297R mutant ORF45 can also be ubiquitylated, it is possible that ubiquitylation of ORF45 at different positions may determine whether ORF45 or ORF45-carrying viral particles bud into Golgi/endosomes vesicles or lysosomes. This is an intriguing issue and warrants further investigation.

Disruption of LRs with MβCD and mutation of Lys297 dramatically reduced virion production, but failed to completely block the virion assembly and release. This suggests that there might be compensated pathways of egress besides the ubiquitin-mediated LR targeting. However, the viral particles released from iSLK-BAC-K297R were less infective, suggesting that other alternative or salvage pathways are not sufficient or efficient to produce mature and infectious viral particles.

**Fig 11:**
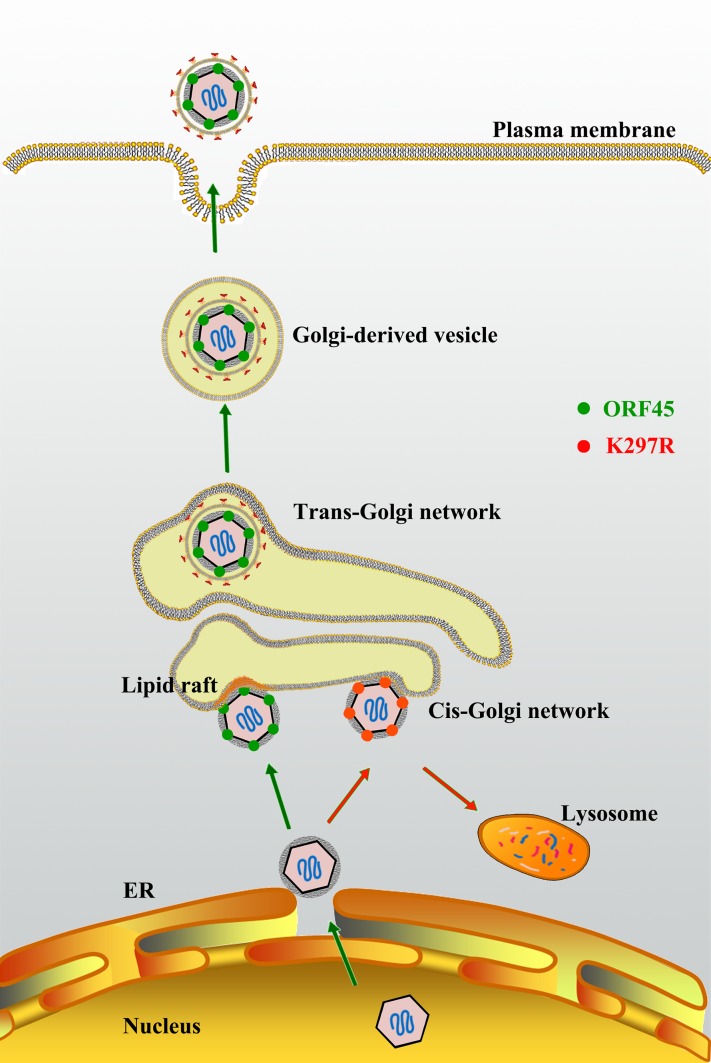
Model for the role of ORF45 in LR-localization and KSHV final envelopment. After budding into the cytoplasm, capsids gain tegument in the cytoplasm. Tegument protein ORF45 directs tegumented capsid targeting LRs for viral assembly in Golgi complex, and budding through Golgi-derived vesicles. LRs serve as a platform for KSHV assembly. Mutation in ORF45 (K297R) results in immature virion particles that fail to target LR, but are degraded in lysosomes.

In summary, our study led to a model for the role of ORF45 in KSHV assembly and egress (Fig 11), which proposes (i) LRs serve as a platform for KSHV assembly, (ii) ORF45 targets LR to direct final envelopment in Golgi-derived vesicles and this process is controlled by ubiquitylation of ORF45 at Lys297, (iii) failure to bud into Golgi-derived vesicles due to mutation of ORF45 at Lys297 results in immature viral particles that are sorted to lysosome for degradation.

## Materials and Methods

### Cells and chemicals

The primary effusion lymphoma cell line BCBL-1, which carries latently infected KSHV and was obtained from the National Institutes of Health AIDS Research and Reference Reagent Program, were grown in RPMI 1640 medium [35]. iSLK cells, that express RTA in a doxycycline-inducible manner [36], and iSLK.219 cells, which harbor rKSHV.219 [37], were cultured in Dulbecco's modified Eagle's medium (DMEM). The iSLK-BAC16 cell line, which carries a BAC-cloned KSHV genome (BAC16), has been described previously [21]. Human embryonic kidney (HEK) 293T cells [38] and HeLa cells were grown in DMEM. All cultures were supplemented with 10% heat-inactivated fetal bovine serum (FBS) and antibiotics.

Doxycycline (Dox), 12-O-tetradecanoylphorbol-13-acetate (TPA), sodium butyrate (NaB), Methyl-β-cyclodextrin (MβCD) and lovastatin were purchased from Sigma-Aldrich (St. Louis, MO). Hygromycin was purchased from Roche (Indianapolis, IN). Turbo DNaseI was obtained from Ambion (Austin, TX).

### Plasmids

The ORF64, ORF65, gH and K8 expressing vectors were described in our previous papers [15, 16], Wild-type KSHV ORF45 or its mutations were cloned into the *Bam*HI and *XhoI* site of the pCMV-3tag-1 vector containing 3×Flag tag.

ORF45(1–296)-Ub was constructed by inserting a fragment of HA-tagged ubiquitin (HA-Ub) between the *HindIII* and *XhoI* sites of pCMV-3tag-1/ORF45(1–296) or pCMV-3tag-1/ORF45(297–300→4A) plasmid, which produces a chimeric protein in which a HA-tagged ubiquitin is fused at the C-terminal of ORF45 truncation 1–296 aa or mutant 297–300→4A. HA-Ub sequence was amplified by PCR with pcDNA3.1/HA-Ub plasmid (Addgene) as a template. Flag-tagged unconjugatable ubiquitin (Ub-G76V) was constructed by inserting a fragment of unconjugatable ubiquitin (Ub-G76V) between the *HindIII* and *XhoI* sites of pCMV-3tag-1 plasmid, Ub-G76V sequence was amplified from “GFP-Ub KO.G76V” plasmid (Addgene) as a template. ORF45-K297R, -K299R or 1-296-Ub were subcloned into the pEGFP-C1 vector (Clontech).

### Antibodies

Mouse anti-flag (M2) and mouse anti-β-actin were purchased from Sigma Aldrich. Mouse anti-ubiquitin was purchased from BD. Rabbit anti-flag, mouse anti-GFP, mouse anti-α-tubulin and rabbit anti-caveolin-1 antibodies were purchased from Cell Signaling. Rabbit anti-TfR antibody was a Proteintech Group product. The rabbit polyclonal antibodies for GM130, EEA1 and LAMP1 were all ABclone products. Rabbit anti-TGN46 was Abcam product, Fluor Alexa-488-conjugated goat anti-mouse IgG, Fluor Alexa-555-conjugated goat anti-mouse/rabbit IgG, and Alexa-647-conjugated goat anti-rabbit IgG were all purchased from Life Technologies. IRDye 680LT and 800CW goat anti-rabbit IgG or anti-mouse IgG antibodies were purchased from LI-COR Biosciences. The mouse monoclonal antibodies against K8, ORF45, ORF64, and ORF65, gH were generated in our lab previously.

### Membrane flotation assay

To isolate detergent-resistant lipid micro-domains (Lipid Rafts), 1×10^7^ HEK293T cells were transiently transfected with ORF45 expression vectors. Forty-eight hour post-transfection, the cells were harvested in pre-cooled NTE buffer (10 mM Tris-HCl [pH7.2], 100 mM NaCl, 1mM EDTA), swollen in 350 μL of hypotonic buffer (10 mM Tris-HCl [pH7.4], 0.2 mM MgCl_2_) at 4°C for 30 min and lysed by passing through a 25G needle. The nuclear debris was removed by centrifugation at 600×g at 4°C. The membrane solution (300 μl) was mixed with Triton X-100 (final concentration 0.5%) and incubated at 4°C for 30 min. Sucrose gradient was made in Beckmen SW55Ti ultracentrifugation tube as follows. Mixture of Triton X-100 treated membrane with 1.8 mL 65% (w/w) sucrose was loaded at the bottom of the tube and then sequentially overlaid with 2.5 mL 45% (w/w) and 1 mL 2.5% (w/w) sucrose solutions. After ultracentrifugation at 200,000×g at 4°C for 16 h, ten fractions (500 μL each) were collected from the top of the tube to the bottom. 20 μL of each fraction was analyzed by Western blotting with antibodies specified for the proteins of interest.

### Cholesterol Depletion and Repletion

In order to deplete cholesterol from cellular membranes, cells were incubated in the presence of 2 μM lovastatin or 1 mM MβCD in DMEM (without FBS) for 1–5 days at 37°C. For cholesterol repletion, the cholesterol depleted cells were washed with serum-free medium and incubated 1–5 days at 37°C in the presence of water-soluble cholesterol (400 μg/mL) and subsequently used for viral DNA extraction.

### Western blotting

Cells were washed with 1×PBS and lysed with cell lysis buffer (50 mM Tris-HCl, pH 7.4, 150 mM NaCl, 1% NP-40, 1 mM sodium orthovanadate [Na3VO4], 20 mM sodium pyrophosphate, 100 mM sodium fluoride, 10% glycerol, protease inhibitor cocktail [1 tablet in 50 mL lysis buffer]). The cell lysates were homogenized and centrifuged at 13,000 rpm for 10 min at 4°C. The whole cell extracts of 50 μg protein was resolved by SDS-PAGE and transferred onto nitrocellulose membranes. The membranes were blocked in 5% non-fat milk in 1×PBS for 1 h, and then incubated in diluted primary antibodies overnight at 4°C. IRDye 680LT and 800CW goat anti-rabbit IgG or anti-mouse IgG antibodies (LI-COR Biosciences) was used as secondary antibody. An Odyssey system (LI-COR) was used for detection of proteins of interest.

### Immunofluorescence assay (IFA)

To detect the subcellular localization of ORF45 and its mutants, iSLK-BAC16 and iSLK-BAC-K297R cells were induced with Dox (3 μg/mL) for 2–3 days. After washing with 1×PBS, cells were fixed with 3.6% formaldehyde in PBS for 10 min, permeabilized in 0.1% Triton X-100 in PBS for 15 min, and blocked in 1% BSA in PBS for 1 h. Then the cells were incubated with mouse anti-ORF45 (made in our lab, 1:200 dilution) and rabbit anti-GM130 (ABclone, 1:100 dilution), anti-EEA1 (ABclone, 1:100 dilution), anti-LAMP1 (ABclone, 1:100 dilution)) or anti-TGN46 (Abcam, 1:500 dilution) antibodies for 1 h. Fluor Alexa-555-conjugated anti-mouse IgG and Fluor Alexa-647-conjugated anti-rabbit IgG (Life technologies, 1:500 dilution) were used as the respective secondary antibodies. The nuclei were stained by Hoechst 33258 (0.1 μg/mL) for 10 min avoid light. Slides were examined with a Zeiss LSM780 confocal laser scanning system (63×oil) and two channels were recorded sequentially. For visualization of the viral particles localization, the viral particles were stained with anti-ORF65 antibody (1:100 dilution).

### Labeling and visualization of lipid rafts with Fluorescent cholera toxin B subunit

Fluor-Alexa-555 conjugated cholera toxin subunit B (CTB-555) (Life Technologies, 1:100 dilution) was used to label GM1 positive LR as previously described [17]. For visualization of LRs on plasma membrane, cells were incubated with CTB-555 for 10–15 min on ice. For visualization of intracellular GM1 positive LRs, cell were incubated at 37°C for 10 min and 60 min, respectively.

To examine the colocalization of ORF45 with dynamic LRs, HeLa cells were transfected with pEGFP/ORF45 and pEGFP/K297R. Forty-eight hour post-transfection, the cells were subjected to CTB-555 labeling as above. To examine the colocalization of LRs with ORF45 and Golgi marker GM-130, iSLK cells were transiently transfected with pCMV-3tag-1/ORF45. Forty-eight hour post-transfection, the cells were subjected to a triple-labeled IFA. CTB labeled LR at 37°C for 30 min, and then the cells were fixed, permeabilized and labeled with mouse-anti-ORF45 and rabbit-anti-GM130 (ABclone) antibodies. Fluor Alexa-488-conjugated anti-mouse IgG and Fluor Alexa-647-conjugated anti-rabbit IgG (Life technologies, 1:500 dilution) were used as the respective secondary antibodies. Cells were examined under a Zeiss LSM780 confocal laser scanning system (63×oil). Images were analyzed by ZEN2009 and Imaris software.

### Genetic manipulation of BAC-cloned KSHV genome

To generate ORF45 K297R mutant virus, mutagenesis of BAC16 was performed using a recombineering system as described in Tischer et al. [22, 39]. In brief, the Kan/I-SceI cassettes were amplified by PCR with pEPKan-S plasmid as a template and the following primers: KS45-K297R-5' (5’-TATTGAGTCAGAGAATCGGGCTCATGGACGTGGGCCAGAGGCGCAAAAGGCAGTCTACCGAGGATGACGACGATAAGTAGGG-3’) and KS45-K297R-3' (5’-ATCCTCGCTACCAGAGGAGGCGGTAGACTGCCTTTTGCGC CTCTGGCCCACGTCCATGAGGCCAGTGTTACAACCAATTAACC-3’). The purified PCR fragments were electroporated into BAC16-containing GS1783 cells that had been induced at 42°C for 15 min. Recombinant clones were selected at 32°C on LB plates containing 34 μg/ml chloramphenicol and 50 μg/ml kanamycin. The resultant colonies were analyzed by restriction enzyme digestion. Positive clones were cultured with 1% L-arabinose, induced at 42°C, and plated on LB plates containing 1% L-arabinose for secondary recombination. Colonies that survived on the L-arabinose plates were replicated on plates with 34 μg/ml chloramphenicol alone and on plates with both 34 μg/ml chloramphenicol and 50 μg/ml kanamycin. Kanamycin-sensitive clones were analyzed by restriction enzyme digestion and proper mutations were further confirmed by DNA sequencing.

### Reconstitution of recombinant KSHVs

iSLK cells seeded in a 24-well plate were transfected with 1 μg of BAC DNAs by Effectene Transfection Reagent (Qiagen). One day post-transfection, cells were subcultured into a T25 flask with fresh medium containing 450 μg/ml G418 and 1μg/ml puromycin. Hygromycin was added to a final concentration of 500 μg/ml next day for selection. After 30 days of selection, hygromycin-resistant colonies were trypsinized, pooled, and subcultured with 1:9 dilution every 3 days.

To induce viral lytic replication, BAC-carrying iSLK cells were seeded into 6-well plate or T150 flask, and 1 day later (when cells reached ~90% confluence), medium was replaced with fresh medium containing 2 μg/ml doxycycline and 1 mM butyrate. Five days post-induction, virion particles were purified from the culture medium. Briefly, the medium was collected and cleared of cells and debris by sequential centrifugation (1000×*g* for 15 min at 4°C and 8000×*g* for 30 min at 4°C). The virions were then pelleted by ultracentrifugation (100,000×*g* for 1 h at 4°C) on a 25% sucrose cushion using a Beckman SW32Ti rotor. The pellets were dissolved in 1/200 of the original volume of 1×PBS or DMEM and stored at -80°C.

### Preparation of intracellular vrial and extracellular virion DNAs

Total DNAs were prepared from cells or viral stocks with a DNeasy tissue kit (QIAGEN). Monolayers of induced iSLK-BAC cells were trypsinized, washed, and resuspended in 200 μL of 1×PBS. Total DNA was prepared according to the manufacturer’s instructions. For the preparation of DNA from intact virions, 200 μl of virus stocks was pretreated with 2 μl of Turbo DNase I (Ambion) for 1 h at 37°C. The reaction was stopped by addition of EDTA followed by heat inactivation at 70°C. Then, 20 μl of proteinase K solution and 200 μl of buffer AL from the DNeasy kit (QIAGEN) were added. The mixture was kept at 70°C for 15 min and then extracted with phenol-chloroform. The DNA was ethanol precipitated with glycogen, and the DNA pellet was dissolved in 40 μl of Tris-EDTA buffer.

### Quantification of viral genomic DNA

Intracellular viral genomic DNA and extracellular virion DNA were extracted from induced iSLK-BAC or iSLK.219 cells or supernatant of cell culture, respectively. Copy numbers of viral genomic DNA were estimated by real-time PCR. The forward primer of LANA (5'-CGCGAATACCGCTATGTACTCA-3') and reverse primer (5'-GGAACGCGC CTCATACGA-3') were described previously [40]. The intracellular viral genomic DNA in each sample was normalized to GAPDH (glyceraldehyde-3-phosphate dehydrogenase) using primers directed to GAPDH (forward, 5’-ACATCATCCCTGCCTCTAC-3’; reverse, 5’-TCAAAGGTGGAGGAGTGG-3’).

### Infection of 293T cells with progeny virus

BAC16 or BAC-K297R virions equivalent to 10^7^ viral DNA copies were employed to infect 10^5^ 293T cell (MOI of 100 viral genomic DNA copies). Cells were incubated with virions in the presence of polybrene (4 μg/ml) in 500 μl DMEM for 5 min in 24-well plate. The plate was then centrifuged at 2000×g for 1 h, and incubated in a CO_2_ incubator. The medium was replaced by fresh DMEM at 6 h post-infection and the cells were cultured for additional 24 h.

The infection rate of 293T cells was examined by fluorescent microscopy and flow cytometry analysis. The infected cells were observed and recorded with a Zeiss LSM780 fluorescent microscope under GFP channel at 24 h post-infection. Subsequently, 1×10^5^ cells were collected and subjected to FACS analysis. The percentage of GFP-positive cells was determined using a FACS CantoII (BD Bioscience). Infection rates are presented as the number of GFP-positive cells in each well and the ratio of GFP-positive cells at the time of the analysis [21].

### Analysis of effects of MβCD on iSLK cells

To assess the cytotoxicity of MβCD, the viabilities of iSLK cells treated with MβCD were determined by counting Trypan blue-stained cells 5 days post-treatment using a light microscope. Cell viabilities were defined relative to control cells (untreated with MβCD). The half-maximal cytotoxic concentration (CC_50_) was calculated from dose-response curves with GraphPad Prism software.

To assess the effect of MβCD on iSLK cell proliferation, 1 × 10^5^ cells were treated with MβCD for 5 days at four different concentrations (0, 0.5, 1, 5 mM) respectively. The cells were collected every day and assayed by MTT.

To assess the effect of MβCD on Lipid raft (LR) or microtubule organization, iSLK cells were treated with 1mM MβCD for 1–5 days and analyzed by IFA. LRs were stained by CTB-555 (Life Technologies, 1:100 dilution) before cell fixation. While, for microtubule staining, the cells were fixed and punched first, and then were stained by mouse anti-α-tubulin (Cell Signaling, 1:100 dilution) and Fluor Alexa-488-conjugated anti-mouse IgG (Life Technologies, 1:500 dilution). The nuclei were stained by Hoechst 33258 (0.1 μg/ml). Slides were examined with a Zeiss LSM780 confocal laser scanning system (63×oil).

## Supporting Information

S1 Table List of genes mentioned in the text and their accession ID numbers. (DOCX)

S1 Fig MβCD Toxicity assessment in iSLK cells. (A) Cytotoxicity of MβCD to iSLK.219 cells was evaluated by treating the cells with a range of concentrations of MβCD for 5 days. The viability of iSLK cells treated with MβCD was assessed by counting Trypan blue-stained cells 5 days post-treatment using a light microscope. Cell viability is defined relative to control cells (no MβCD treated). The half-maximal cytotoxic concentration (CC_50_ = 4.627 mM) was calculated from dose-response curves with GraphPad Prism software. (B) Effect of MβCD on iSLK cell proliferation. iSLK cells (starting with 1×10^5^ cells/ml) were exposed to MβCD at indicated concentrations and subjected to MTT every day for 5 days. Data were obtained from three independent determinations and are presented as means with standard deviations. (C) Lipid rafts in iSLK cells, treated or not with 1mM MβCD for 1 day, were stained with CTB-555. (D) iSLK cells, treated with 1mM MβCD for 5 days, were stained by anti-α-tubulin for microtubules (green) at the indicated time points. The nuclei were stained by Hoechst (blue).
(PDF)

S2 Fig Lovastatin suppresses KSHV virion production in iSLK.219 cells. iSLK.219 cells were induced by Doxycycline (Dox) for KSHV lytic replication in the absence and presence of 2 μM Lovastatin (Lov). The cells and culture medium were collected at the indicated time (days). The extracellular virion DNA copy number and intracellular viral genomic DNA were quantitated by qPCR as described in Materials and Methods. (*, *p*< 0.05).
(PDF)

S3 Fig Localization of ORF45 and its mutants (K297R and K299R) in HeLa cells. GFP-tagged ORF45, K297R and K299R were introduced into HeLa cells by transfcetion, respectively. Forty-eight hour post-transfection, the cells were fixed and the nuclei were stained by Hoechst. The localization of ORF45 and its mutants were examined under a Zeiss LSM780 confocal laser scanning system (63×oil).
(PDF)

S4 Fig Colocalization of ORF45 with lipid rafts was suppressed by unconjugatable ubiquitin (Ub-G76V). Ub-G76V was introduced into HeLa cells by transfection with GFP-tagged ORF45 and 1-296-Ub expression vectors, respectively. Forty-eight hour post-transfection, live HeLa cells were stained by CTB-555 (red) as described in Materials and Methods. The colocalization of ORF45-GFP and lipid rafts was examined by merging images from two channels (Panels C, F, I and L). Images were captured under a Zeiss LSM780 confocal laser scanning system (63×oil).
(PDF)

S5 Fig Construction and characterization of a recombinant KSHV carrying an ORF45 K297R mutant on BAC16. (A) The structure and chromatograms of BAC DNAs at ORF45 locus. The upper panel shows the KpnI as well as HindIII restriction site around ORF45 on BAC-cloned KSHV genome. The sequence modification in ORF45 around the point mutation from AAG to AGG in K297R mutant is shown below the DNA structure. (B) Sequences of the wild type and mutant BACs at the ORF45 locus. The sequence chromatogram and deduced amino acids are shown. The designed mutation is boxed. (C) Restriction enzyme digestion of purified KSHV BAC DNAs with Kpn I or Hind III. No nonspecific or spurious rearrangements were observed in any of the mutant BAC.
(PDF)

S6 Fig Colocalization of ORF45 and ORF65 in iSLK cells. iSLK-BAC16 and iSLK-BAC-K297R cells, induced with Dox (3 μg/ml) for 3 days, were subject to IFA stained with rabbit polyclonal anti-ORF45 (green) and mouse polyclonal anti-ORF65 (red). The nuclei were stained by Hoechst (blue). Three channels merged images are shown in Panels D and P, respectively. The white boxes within the merged panels are shown as enlarged pictures for the details of colocalization.
(PDF)

S1 Video Dynamics of GM1-positive lipid raft endocytosis and colocalization with ORF45 visualized in live cells. HeLa cells were transfected with the expression vector of GFP-tagged ORF45 (Green). Forty-eight hour post-transfection, live cells were stained with CTB-555 (Red) and video-recorded for 30 min under a Zeiss LSM780 confocal laser scanning system (63×oil).
(AVI)

S2 Video Effect of MβCD treatment on lipid raft endocytosis and colocalization with ORF45 in live cells. HeLa cells were transfected with the expression vector of GFP-tagged ORF45 (Green). Twenty-four hour post-transfection, live cells were treated with 1 mM MβCD for 24 h. Then cells were stained with CTB-555 (Red) and video-recorded for 30 min under a Zeiss LSM780 confocal laser scanning system (63×oil).
(AVI)
