# Supplementary material for: Mono-ubiquitylated ORF45 Mediates Association of KSHV Particles with Internal Lipid Rafts for Viral Assembly and Egress
Source: PLoS Pathog. 2015 Dec 9;11(12):e1005332. doi: 10.1371/journal.ppat.1005332 (PMC4674120; doi:10.1371/journal.ppat.1005332)
Supplement: S1 Table — (DOCX) [file ppat.1005332.s001.docx]

**Table S1.** List of genes mentioned in the text and their accession ID numbers.

| Gene name | GENE ID |
| --- | --- |
| Caveolin-1 | 857 |
| EEA-1 | 8411 |
| GM130 | 2801 |
| KSHV LANA | 4961527 |
| KSHV K8 | 4961462 |
| KSHV ORF22 (gH) | 4961506 |
| KSHV ORF45 | 4961474 |
| KSHV ORF64 | 4961441 |
| KSHV ORF65 | 4961451 |
| LAMP-1 | 3916 |
| TfR (Transferrin receptor) | 7037 |
| TGN46 | 10618 |
| Ubiquitin | 7314 |
