# Supplementary material for: Mono-ubiquitylated ORF45 Mediates Association of KSHV Particles with Internal Lipid Rafts for Viral Assembly and Egress
Source: PLoS Pathog. 2015 Dec 9;11(12):e1005332. doi: 10.1371/journal.ppat.1005332 (PMC4674120; doi:10.1371/journal.ppat.1005332)
Supplement: S1 Fig — (A) Cytotoxicity of MβCD to iSLK.219 cells was evaluated by treating the cells with a range of concentrations of MβCD for 5 days. The viability of iSLK cells treated with MβCD was assessed by counting Trypan blue-stained cells 5 days post-treatment using a light microscope. Cell viability is defined relative to control cells (no MβCD treated). The half-maximal cytotoxic concentration (CC50 = 4.627 mM) was calculated from dose-response curves with GraphPad Prism software. (B) Effect of MβCD on iSLK cell proliferation. iSLK cells (starting with 1×10^5^ cells/ml) were exposed to MβCD at indicated concentrations and subjected to MTT every day for 5 days. Data were obtained from three independent determinations and are presented as means with standard deviations. (C) Lipid rafts in iSLK cells, treated or not with 1mM MβCD for 1 day, were stained with CTB-555. (D) iSLK cells, treated with 1mM MβCD for 5 days, were stained by anti-α-tubulin for microtubules (green) at the indicated time points. The nuclei were stained by Hoechst (blue). (PDF) [file ppat.1005332.s002.pdf]

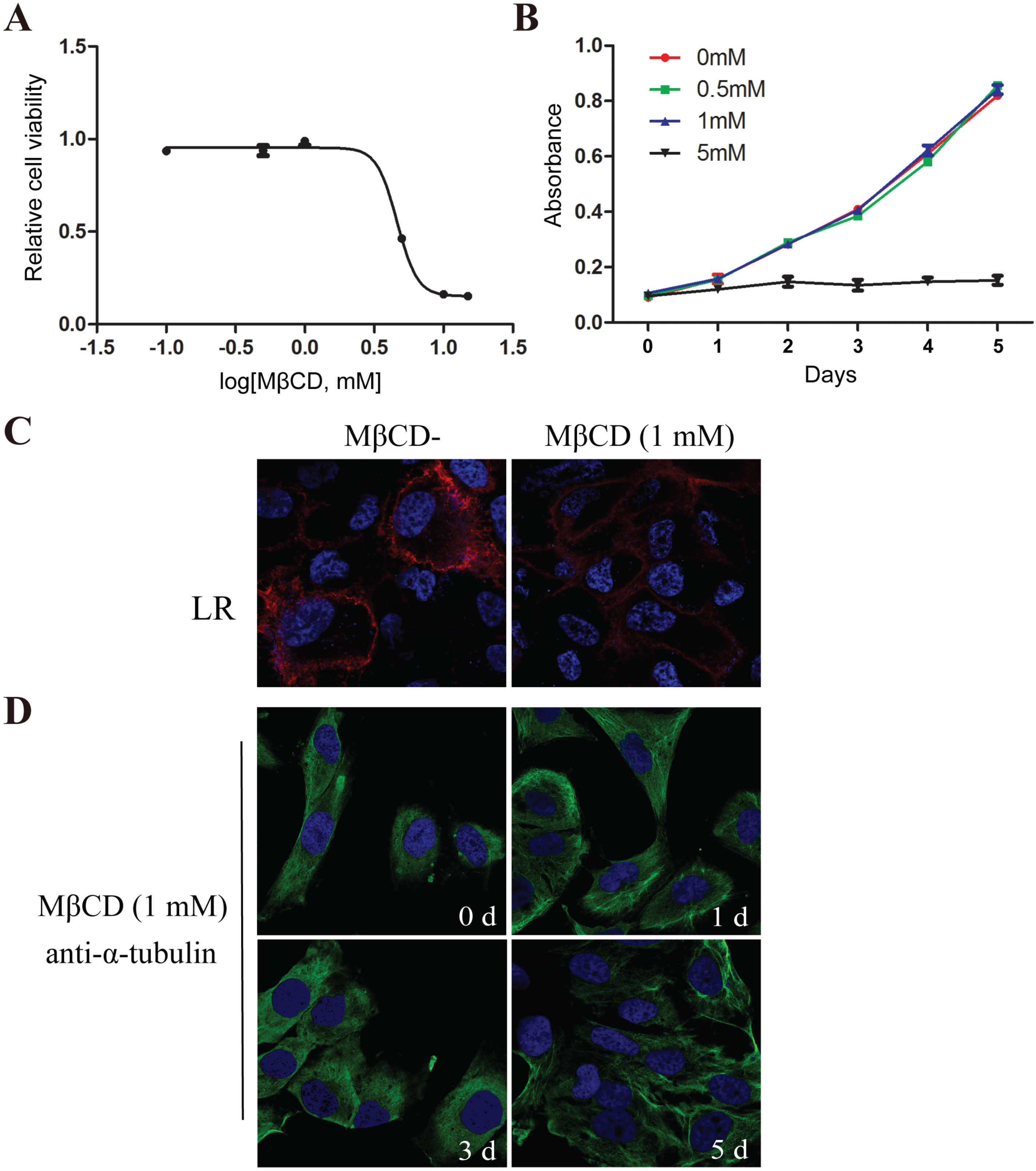

Figure S1. MβCD Toxicity assessment in iSLK cells. (A) Cytotoxicity of MβCD to iSLK.219 cells was evaluated by treating the cells with a range of concentrations of MβCD for 5 days. The viability of iSLK cells treated with MβCD were assessed by counting Trypan blue-stained cells 5 days post-treatment under a light microscope. Cell viability is defined relative to control cells (no MβCD treated). The half-maximal cytotoxic concentration (CC_{50} = 4.627 mM) was calculated from dose-response curves with GraphPad Prism software. (B) Effect of MβCD on iSLK cell proliferation. The iSLK cells (starting with 1×10^5 cells/ml) were exposed to MβCD at indicated concentrations and subjected to MTT every day for 5 days. Data were obtained from three independent determinations and are presented as means with standard deviations. (C) Lipid rafts in iSLK cells, treated with or without 1mM MβCD for 1 day, was stained with CTB-555. (D) iSLK cells, treated with 1mM MβCD for 5 days, were stained by anti-α-tubulin for microtubules (green) at the indicated time points. The nuclei were stained by Hoechst (blue).
