# Supplementary material for: Mono-ubiquitylated ORF45 Mediates Association of KSHV Particles with Internal Lipid Rafts for Viral Assembly and Egress
Source: PLoS Pathog. 2015 Dec 9;11(12):e1005332. doi: 10.1371/journal.ppat.1005332 (PMC4674120; doi:10.1371/journal.ppat.1005332)
Supplement: S2 Fig — iSLK.219 cells were induced by Doxycycline (Dox) for KSHV lytic replication in the absence and presence of 2 μM Lovastatin (Lov). The cells and culture medium were collected at the indicated time (days). The extracellular virion DNA copy number and intracellular viral genomic DNA were quantitated by qPCR as described in Materials and Methods. (*, p< 0.05). (PDF) [file ppat.1005332.s003.pdf]

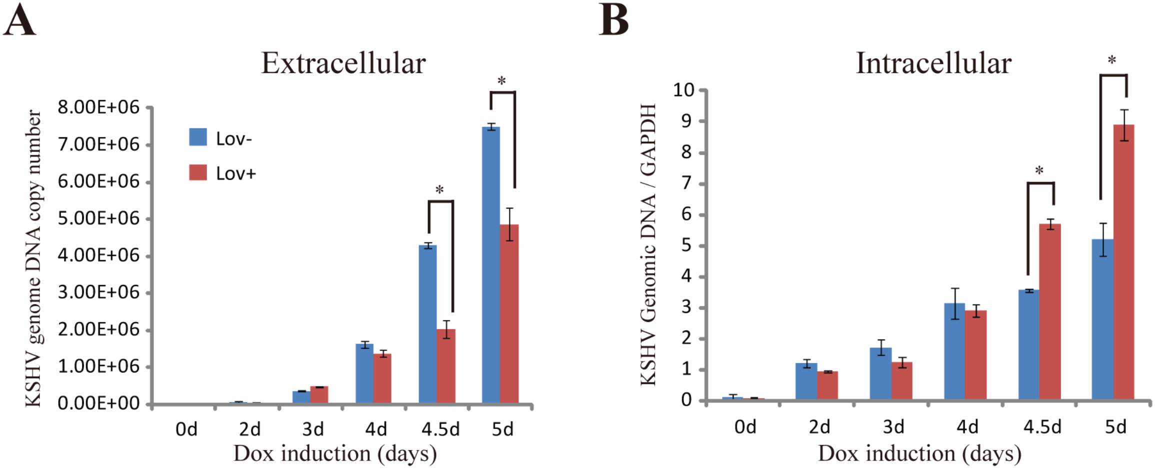

Figure S2. Lovastatin suppresses KSHV virion production in iSLK.219 cells. iSLK.219 cells were induced by Doxycycline (Dox) for KSHV lytic replication in the absence and presence of 2 µM Lovastatin (Lov). The cells and culture medium were collected at the indicated time (days). The extracellular virion DNA copy number and intracellular viral genomic DNA were quantitated by qPCR as described in Materials and Methods. (*, $p < 0.05$).
