# Supplementary material for: Mono-ubiquitylated ORF45 Mediates Association of KSHV Particles with Internal Lipid Rafts for Viral Assembly and Egress
Source: PLoS Pathog. 2015 Dec 9;11(12):e1005332. doi: 10.1371/journal.ppat.1005332 (PMC4674120; doi:10.1371/journal.ppat.1005332)
Supplement: S3 Fig — GFP-tagged ORF45, K297R and K299R were introduced into HeLa cells by transfcetion, respectively. Forty-eight hour post-transfection, the cells were fixed and the nuclei were stained by Hoechst. The localization of ORF45 and its mutants were examined under a Zeiss LSM780 confocal laser scanning system (63×oil). (PDF) [file ppat.1005332.s004.pdf]

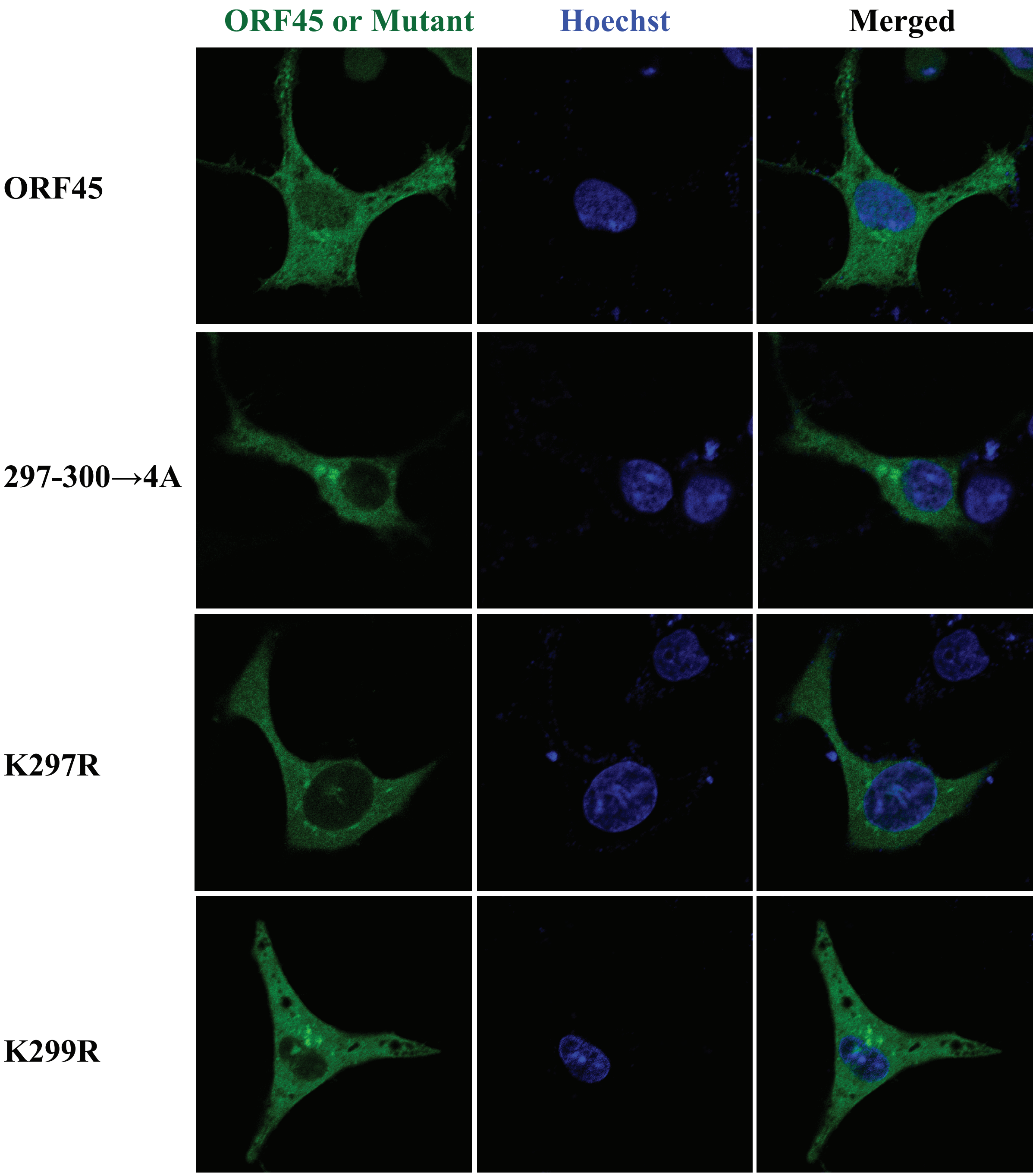

Figure S3. Localization of ORF45 and its mutants K297R and K299R in HeLa cells. GFP-tagged ORF45, K297R and K299R were introduced into Hela cells by transfcetion, respectively. Forty-eight hour post-transfection, the cells were fixed and the nuclei were stained by Hoechst. The localization of GFP-tagged proteins was examined under a Zeiss LSM780 confocal laser scanning system (63×oil).
