# Supplementary material for: Mono-ubiquitylated ORF45 Mediates Association of KSHV Particles with Internal Lipid Rafts for Viral Assembly and Egress
Source: PLoS Pathog. 2015 Dec 9;11(12):e1005332. doi: 10.1371/journal.ppat.1005332 (PMC4674120; doi:10.1371/journal.ppat.1005332)
Supplement: S4 Fig — Ub-G76V was introduced into HeLa cells by transfection with GFP-tagged ORF45 and 1-296-Ub expression vectors, respectively. Forty-eight hour post-transfection, live HeLa cells were stained by CTB-555 (red) as described in Materials and Methods. The colocalization of ORF45-GFP and lipid rafts was examined by merging images from two channels (Panels C, F, I and L). Images were captured under a Zeiss LSM780 confocal laser scanning system (63×oil). (PDF) [file ppat.1005332.s005.pdf]

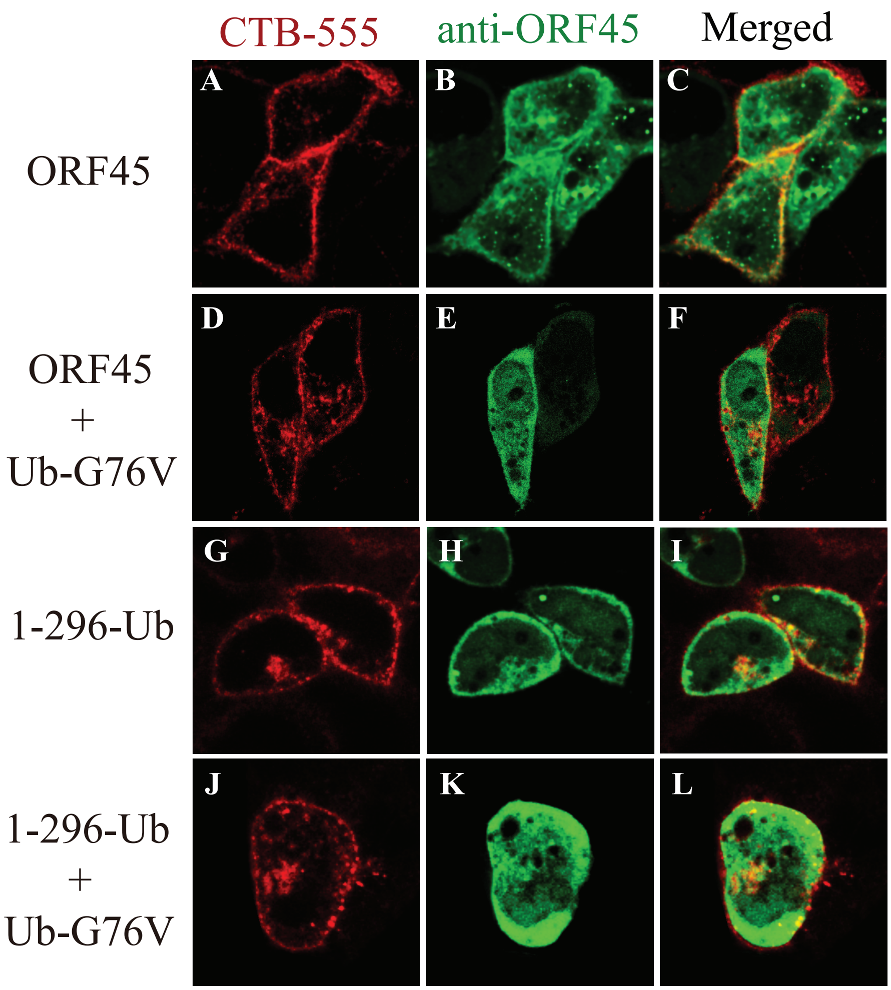

Figure S4. Colocalization of ORF45 with lipid rafts was suppressed by unconjugatable ubiquitin (Ub-G76V). Ub-G76V was introduced into HeLa cells by transfection with GFP-tagged ORF45 and 1-296-Ub expression vectors, respectively. Forty-eight hour post-transfection, live HeLa cells were stained by CTB-555 (red) as described in Materials and Methods. The colocalization of ORF45-GFP and lipid rafts was examined by merging images in two channels (Panels C, F, I and L). Images were captured under a Zeiss LSM780 confocal laser scanning system (63×oil).
