# Supplementary material for: Mono-ubiquitylated ORF45 Mediates Association of KSHV Particles with Internal Lipid Rafts for Viral Assembly and Egress
Source: PLoS Pathog. 2015 Dec 9;11(12):e1005332. doi: 10.1371/journal.ppat.1005332 (PMC4674120; doi:10.1371/journal.ppat.1005332)
Supplement: S5 Fig — (A) The structure and chromatograms of BAC DNAs at ORF45 locus. The upper panel shows the KpnI as well as HindIII restriction site around ORF45 on BAC-cloned KSHV genome. The sequence modification in ORF45 around the point mutation from AAG to AGG in K297R mutant is shown below the DNA structure. (B) Sequences of the wild type and mutant BACs at the ORF45 locus. The sequence chromatogram and deduced amino acids are shown. The designed mutation is boxed. (C) Restriction enzyme digestion of purified KSHV BAC DNAs with Kpn I or Hind III. No nonspecific or spurious rearrangements were observed in any of the mutant BAC. (PDF) [file ppat.1005332.s006.pdf]

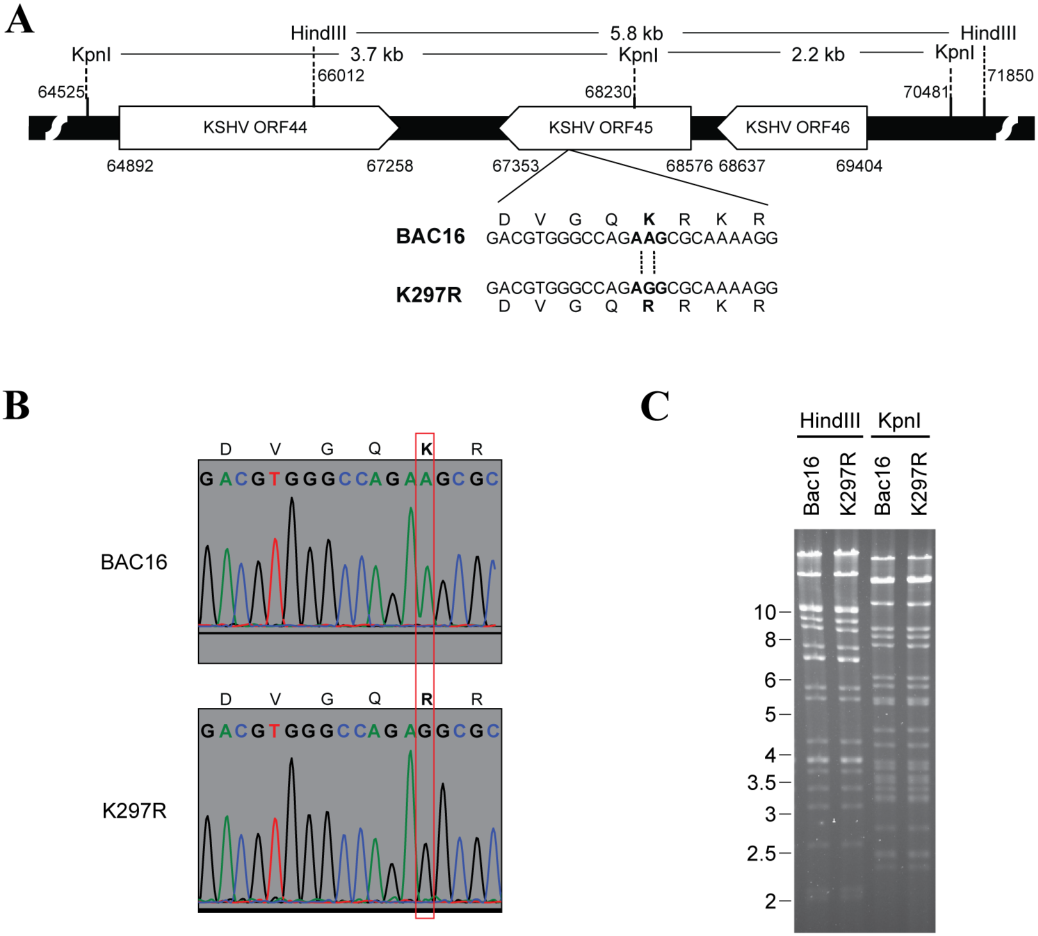

Figure S4. Construction and analyses of a recombinant KSHV carrying an ORF45 K297R mutant. (A) The structure and chromatograms of BAC DNAs at ORF45 locus. The upper panel shows the KpnI as well as HindIII restriction site around ORF45 on BAC-clonedKSHV genome (BAC16). The sequence modification in ORF45 around the point mutation from AAG to AGG in K297R mutant is shown below the DNA structure. (B) Sequences of the wild type and mutant BACs at the ORF45 locus. The sequence chromatogram and deduced amino acids are shown. The designed mutation is boxed. (C) Restriction enzyme digestion of purified KSHV BAC DNAs with KpnI or HindIII. No nonspecific or spurious rearrangements were observed in any of the recombinant viruses.
