# Supplementary material for: Mono-ubiquitylated ORF45 Mediates Association of KSHV Particles with Internal Lipid Rafts for Viral Assembly and Egress
Source: PLoS Pathog. 2015 Dec 9;11(12):e1005332. doi: 10.1371/journal.ppat.1005332 (PMC4674120; doi:10.1371/journal.ppat.1005332)
Supplement: S6 Fig — iSLK-BAC16 and iSLK-BAC-K297R cells, induced with Dox (3 μg/ml) for 3 days, were subject to IFA stained with rabbit polyclonal anti-ORF45 (green) and mouse polyclonal anti-ORF65 (red). The nuclei were stained by Hoechst (blue). Three channels merged images are shown in Panels D and P, respectively. The white boxes within the merged panels are shown as enlarged pictures for the details of colocalization. (PDF) [file ppat.1005332.s007.pdf]

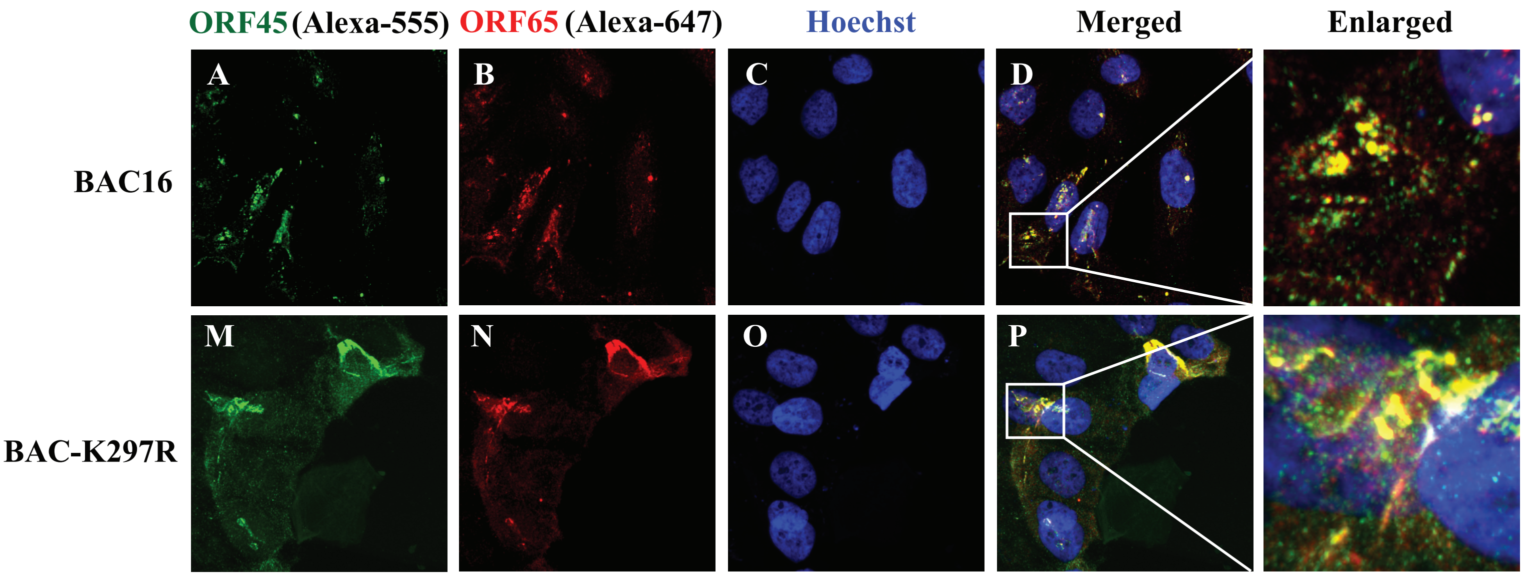

Figure S6. Colocalization of ORF45 and ORF65 in iSLK cells. iSLK-BAC16 and iSLK-BAC-K297R cells, induced with Dox (3 µg/ml) for 3 days, were subject to IFA stained with rabbit polyclonal anti-ORF45 (green) and mouse polyclonal anti-ORF65 (red). The nuclei were stained by Hoechst (blue). The three channels merged images are shown in Panels D and P, respectively. The white boxes within the merged panels are shown as enlarged pictures for the details of colocalization.
